# Disruption of DNA repair in cancer cells by ubiquitination of a destabilising dimerization domain of nucleotide excision repair protein ERCC1

**DOI:** 10.18632/oncotarget.19422

**Published:** 2017-07-21

**Authors:** Lanlan Yang, Ann-Marie Ritchie, David W. Melton

**Affiliations:** ^1^ Edinburgh Cancer Research Centre, MRC Institute of Genetics and Molecular Medicine, University of Edinburgh, Western General Hospital, Edinburgh, EH4 2XU, UK

**Keywords:** nucleotide excision repair, ERCC1-XPF, ERCC1 homodimerization, ubiquitination, destabilisation

## Abstract

DNA repair pathways present in all cells serve to preserve genome stability, but in cancer cells they also act reduce the efficacy of chemotherapy. The endonuclease ERCC1-XPF has an important role in the repair of DNA damage caused by a variety of chemotherapeutic agents and there has been intense interest in the use of ERCC1 as a predictive marker of therapeutic response in non-small cell lung carcinoma, squamous cell carcinoma and ovarian cancer. We have previously validated ERCC1 as a therapeutic target in melanoma, but all small molecule ERCC1-XPF inhibitors reported to date have lacked sufficient potency and specificity for clinical use. In an alternative approach to prevent the repair activity of ERCC1-XPF, we investigated the mechanism of ERCC1 ubiquitination and found that the key region was the C-terminal (HhH)_2_ domain which heterodimerizes with XPF. This ERCC1 region was modified by non-conventional lysine-independent, but proteasome-dependent polyubiquitination, involving Lys33 of ubiquitin and a linear ubiquitin chain. XPF was not polyubiquitinated and its expression was dependent on presence of ERCC1, but not vice versa. To our surprise we found that ERCC1 can also homodimerize through its C-terminal (HhH)_2_ domain. We exploited the ability of a peptide containing this C-terminal domain to destabilise both endogenous ERCC1 and XPF in human melanoma cells and fibroblasts, resulting in reductions of up to 85% in nucleotide excision repair and near two-fold increased sensitivity to DNA damaging agents. We suggest that the ERCC1 (HhH)_2_ domain could be used in an alternative strategy to treat cancer.

## INTRODUCTION

As a key endonuclease in a major DNA repair pathway, the role of ERCC1-XPF in the development of ageing [1, 2], neurodegenerative disease [3] and especially in multiple forms of cancer (reviewed in [4]) has been widely investigated. Despite the success of new targeted cancer therapies, DNA-damaging chemotherapeutics remain the current mainstay of treatments for the majority of common cancers. DNA repair pathways present in all cells serve to preserve genome stability, but in cancer cells they also act to reduce the efficacy of chemotherapy. ERCC1-XPF has an important role in the repair of DNA damage caused by a variety of chemotherapeutic agents. It is essential for the nucleotide excision repair (NER) pathway that removes helix-distorting lesions caused by UV irradiation, bulky alkylating agents and intrastrand cross-linkers. It also has an important role in removal of the particularly toxic, and so therapeutically very effective, interstrand crosslinks (ICLs, reviewed in [4]) Since the most commonly used cancer chemotherapeutics, platinating agents such as cisplatin and carboplatin, cause all three types of lesion, there has been intense interest in the use of ERCC1 as a predictive marker of therapeutic response to these agents.

High ERCC1 expression at the mRNA or protein level has been linked in many studies with poor response to chemotherapy: non-small cell lung carcinoma [5–8]; squamous cell carcinoma [9, 10]; and ovarian cancer [11]. However, this correlation was not universal [12–14] and lack of specificity of many of the antibodies used for immunohistochemical detection of ERCC1 has been documented [15]. Lack of correlation between ERCC1 mRNA and protein levels in ovarian cancers has also been reported [11], suggesting that there is not a simple direct relationship between ERCC1 mRNA levels and DNA repair capacity.

Additional complications in the use of ERCC1-XPF as a predictive marker of response are the need to carefully regulate the endonuclease activity of ERCC1-XPF to prevent activity on non-damaged DNA and the requirement to function as a heterodimer. ERCC1 is principally responsible for recruitment to repair foci and XPF provides the endonuclease activity (reviewed in [4]). Previous observations indicated that ERCC1 and XPF molecules outside ERCC1-XPF heterodimers are rapidly degraded [16]. Xeroderma pigmentosum patients belonging to the XPF complementation group have reduced cellular levels of both XPF and ERCC1 and, conversely, ERCC1-deficient cells show low levels of XPF, implying that formation of the heterodimeric complex stabilises both proteins *in vivo* [17, 18]. The key protein-protein interaction between ERCC1 and XPF is dimerization of the hydrophobic C-terminal regions through their double helix-hairpin-helix (HhH_2_) domains [19, 20]. Without heterodimerization it was conventionally thought neither protein was stable and that, following exposure of their hydrophobic interaction regions, each was rapidly degraded [20, 21]. However, more recent siRNA experiments have indicated that, while XPF protein levels were decreased when ERCC1 was knocked down, the converse was not true [22].

We have previously validated ERCC1 as a therapeutic target by showing that a genetically engineered ERCC1-deficient mouse model of melanoma was uniquely sensitive to the chemotherapeutic cisplatin [23]. Inhibitors of the ERCC1/XPA interaction with activity against colorectal and lung cancer cell lines have been reported [24, 25] and an *in silico* drug screen identified compounds that can disrupt ERCC1-XPF complex stability in cell lysates [26]. However, despite the availability of structural information and much endeavour, these studies and our own efforts [27–29] had been unable to identify ERCC1-XPF inhibitors of sufficient potency and specificity in preclinical models to cause the major reductions in NER activity and increased sensitivity to DNA damaging agents needed to justify further development.

Instead we chose to investigate the relationship between ERCC1-XPF and the response to cisplatin in melanoma -a cancer notoriously resistant to chemotherapy. A study of six human melanoma cell lines (Yang and Melton, unpublished) showed that, although mRNA levels of both ERCC1 and XPF were increased by cisplatin treatment as we had reported previously [30], the correlation with ERCC1 and XPF protein levels varied between the cell lines, raising the possibility that post-translational modification may also play an important role in the regulation of ERCC1-XPF repair activity. Since ubiquitin modification is involved in the regulation of both DNA repair activity and DNA repair protein levels (reviewed in [31]), we chose to investigate the detailed mechanism of ERCC1 ubiquitination. During the course of this investigation reported here we also discovered that, contrary to conventional understanding, ERCC1 can also form homodimers in human cells. We further exploited a peptide containing this dimerization domain to destabilise both endogenous ERCC1 and XPF, resulting in major reductions in nucleotide excision repair and increased sensitivity to DNA damaging agents in melanoma cells. These results suggest that this approach could be developed into a viable therapeutic alternative to small molecule inhibitors.

## RESULTS

### The proteasome system is involved in the stability of ERCC1 but not XPF

To investigate the possibility that ubiquitin modification is involved in the regulation of ERCC1 and XPF, we used western blotting to assess the response of both proteins from two human melanoma cell lines, A375 (from a metastatic melanoma) and C32 (from a primary melanoma), to proteasome inhibitor MG132 and translation inhibitor cycloheximide (Supplementary Figure 1). In both cell lines exposure to MG132 caused a time-dependent increase in levels of ERCC1, but not of XPF. In cycloheximide-exposed samples, ERCC1 showed a time-dependent decrease, while XPF again showed no clear change. We concluded that the ubiquitin proteasome system may be involved in the stability of ERCC1, but not XPF.

### ERCC1 is modified by polyubiquitination involving Lys 33 of ubiquitin and by linear polyubiquitination

**Figure 1:**
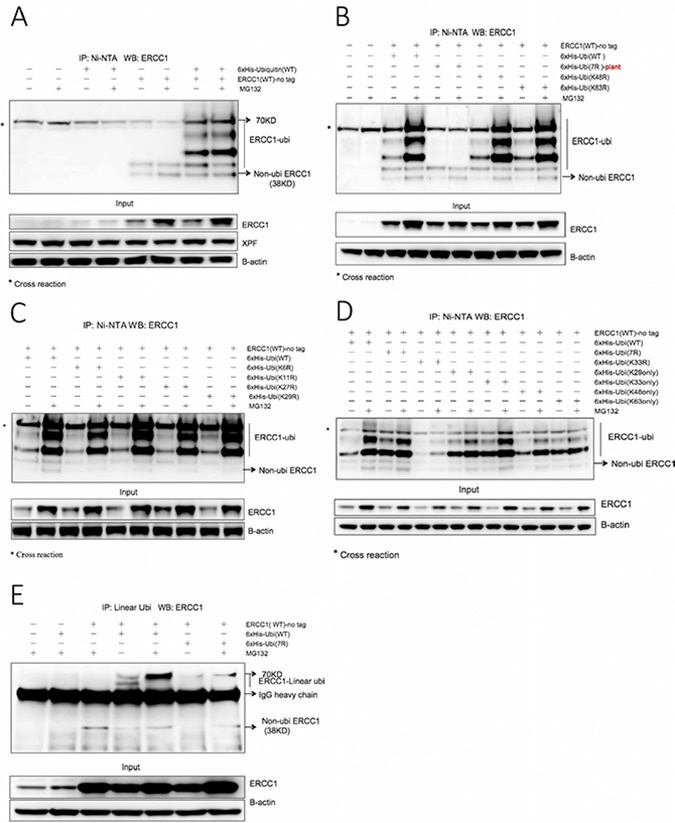
ERCC1 is modified by ubiquitination involving both ubiquitin Lys 33 and linear polyubiquitination (**A**) 6xHis-tagged wild-type (WT) human ubiquitin and non-tagged wild-type human ERCC1 were co-transfected into A375 cells in duplicate 60 mm dishes, while non-transfected, ubiquitin (WT) transfected-only and ERCC1 (WT) transfected-only duplicate dishes served as controls. After 24 h, one dish from each pair was exposed to MG132 (25 μΜ) for 5 h and the *in vivo* ubiquitination assay was then performed. Material purified on Ni beads was western blotted for ERCC1. The ubiquitinated ERCC1 ladder, extending to 70 kD is identified, as is the position of non-ubiquitinated ERCC1 at 38 kD (unmodified ERCC1 consistently runs at this position although the predicted mol wt of the 297 amino acid protein is 32.57 kD). *, denotes a cross-reacting band, also at 70 kD, present in all samples. A sample of the input material prior to purification was also blotted for ERCC1, XPF and beta-actin. Note the difference between low endogenous ERCC1 expression only in the first four lanes of the input blot and high vector-derived plus endogenous ERCC1 expression in the rest of the gel. (**B**) Non-tagged wild-type ERCC1 was transfected alone into A375 cells, or co-transfected with one of four different 6xHis-tagged ubiquitin constructs: wild-type human ubiquitin, plant (*Arabidopsis thaliana*) ubiquitin 7R (where all 7 Lysines were mutated to Arginines), human ubiquitin point mutants (K48R) and (K63R). Dishes were then treated and processed as for panel A. Note the lack of ubiquitination ladder with the plant ubiquitin (7R) mutant and that ubiquitin (K48R) and (K63R) mutants give the same ubiquitination ladder as wild-type ubiquitin. (**C**) Non-tagged wild-type ERCC1 was co-transfected into A375 cells with a 6xHis-tagged human wild-type, or point mutant ubiquitin construct: ubiquitin (K6R), ubiquitin (K11R), ubiquitin (K27R), ubiquitin (K29R). Dishes were then treated and processed as for panel A. (**D**) Non-tagged wild-type ERCC1 was co-transfected into A375 cells with 6xHis-tagged human wild-type, or the human ubiquitin (7R) multiple mutant, or point mutant ubiquitin (K33R), or a combination mutant: ubiquitin (K29 only), ubiquitin (K33 only), ubiquitin (K48 only), ubiquitin (K63 only). Dishes were then treated and processed as for panel A. Note the altered ubiquitination pattern with the (K33R) mutant. Note the stronger ubiquitination with ubiquitin (K33 only) after MG132 treatment compared with the other three combination mutants. Note also the strong ubiquitination pattern with human ubiquitin (7R), compared to the plant ubiquitin (7R) mutant in panel B. (**E**) To investigate linear polyubiquitination, non-tagged wild-type ERCC1 was co-transfected into A375 cells in duplicate 100 mm dishes with 6xHis-tagged human wild-type ubiquitin or ubiquitin (7R). After 24 h, one dish from each pair was exposed to MG132 (25 μΜ) for 5 h. Non-transfected, ubiquitin (WT) transfected-only and ERCC1 (WT) transfected-only dishes, all exposed to MG132, were used as controls. Immunoprecipitation was performed on cell extracts with an antibody specific for linear polyubiquitination and collected with magnetic protein A beads. Material eluted from the beads was western blotted for ERCC1. The position of ERCC1 with linear polyubiquitin chains is indicated, as is the position of heavy chain immunoglobulin. A sample of the input material prior to immunopurification was also blotted for ERCC1 and beta-actin. Note the linear polyubiquitination ERCC1 bands in the co-transfection with wild-type ubiquitin with the stronger signal after MG132 exposure, and the weaker signal in the co-transfection with ubiquitin (7R).

To investigate ubiquitination of ERCC1, ubiquitination assays were performed following overexpression in A375 melanoma cells of non-tagged wild-type human ERCC1 and a series of 6xHis-tagged wild-type human ubiquitin constructs, with or without exposure to MG132 (Figure 1). 10% of cells from each sample were lysed as the input sample, the remainder were extracted and purified on Ni-NTA-agarose beads as the IP sample. Overexpression of ERCC1 was clearly visible in the input sample blot, with a further increase following exposure to MG132, but no clear change in levels of endogenous XPF was seen (Figure 1A). In the IP sample blot, compared with the non-transfected control and 6xHis-tagged ubiquitin transfected-only control, the 6xHis-tagged ubiquitin and non-tagged ERCC1 co-transfected samples showed multiple bands, indicating polyubiquitination or multiple monoubiquitination of ERCC1. The co-transfected MG132-exposed sample showed a stronger ubiquitin ladder. Current understanding of proteasome-dependent protein degradation is that the substrate is first polyubiquitinated and then degraded by the 26S proteasome, containing a 20S catalytic unit and two 19S regulatory units, or alternatively substrate may be degraded in a ubiquitin-independent manner by a proteasome containing the 20S catalytic unit and different regulator units, such as 11S [32, 33]. So we interpret our result to indicate that ERCC1 is more likely to be polyubiquitinated. By rerunning IP and input samples together on the same gel, we showed that the bottom rung of the ubiquitinated ERCC1 ladder comigrated with the 38 kD ERCC1 input monomer (Supplementary Figure 2A). Interpretation of the number of ubiquitins (an 8.5 kD monomer) added to ERCC1 in the co-transfected samples was complicated by the result in the two non-tagged ERCC1 transfected-only control lanes. In addition to the cross-reacting band at 70 kD present in all IP samples, these lanes contained two additional bands also present in the co-transfected samples. The smaller band at 38 kD comigrated with non-modified ERCC1, with the size of the larger band consistent with ERCC1 modified by one ubiquitin. These bands may be present as a result of some non-specific binding of overexpressed ERCC1 to the beads. For the co-transfected panel, the sizes of the other three bands are consistent with ERCC1 modified by two, three and four ubiquitins. Endogenous XPF was unaffected by any of these treatments.

We next investigated whether either of the two most common ubiquitin linkages regulating protein stability, Lys48 (proteasome-mediated proteolysis, [34]) and Lys63 (lysosome-mediated proteolysis, [35]) were involved in ERCC1 ubiquitination (Figure 1B). Non-tagged ERCC1 was co-transfected with 6xHis-tagged wild-type human ubiquitin, plant ubiquitin 7R where all 7 lysines were mutated to arginine (initially only an *Arabidopsis thaliana*, rather than a human 7R construct was available), human ubiquitin K48R (Lys48 mutated to Arg) and ubiquitin K63R. Ubiquitin mutants K48R and K63R showed the same ubiquitination ladder as the wild type, with increased intensity in MG132-exposed samples. As expected, the ubiquitination ladder was absent from the plant ubiquitin (7R) samples. We conclude that ERCC1 is not regulated through Lys48- or Lys63- linked ubiquitination. Therefore, the role of additional ubiquitin lysines (K6, K11, K27, K29) was investigated in the same way (Figure 1C). All mutants showed the identical ubiquitination ladder as the wild type. Supplementary Figure 2B shows the entire gel run on these IP samples, revealing a weak ubiquitination smear, extending above the discrete four-runged ubiquitination ladder in all MG132-exposed samples.

The remaining ubiquitin lysine mutant K33R was investigated together with ubiquitin combination mutants (29K only), (33K only), (48K only) and (63K only), where the remaining six lysines in each construct were all mutated to arginine. A new human, rather than plant, ubiquitin (7R) negative control was also used (Figure 1D). Unlike all of the other point mutants, K33R gave a much weaker ubiquitination ladder than wild-type ubiquitin. All four combination mutants tested gave ubiquitination ladders, but only the ubiquitin (33K only) mutant gave a ladder intensity equivalent to wild type. We conclude that K33 of ubiquitin has a role in, but is not sufficient for, proteasome dependent ubiquitination of ERCC1. Intriguingly, in negative control human ubiquitin (7R) samples, which were expected to lack a ubiquitin ladder in the same way as with the plant ubiquitin (7R) in Figure 1B, the ubiquitin ladder was as strong as for wild-type ubiquitin. This could be due to linear polyubiquitination involving an N-Met, rather than an internal lysine linkage [36]. The failure to see this with the plant (7R) mutant could result from an altered structure due to the three amino acid differences from human ubiquitin.

To investigate if linear polyubiquitination of ERCC1 occurs, conventional immunoprecipitation with a linear polyubiquitin-specific monoclonal antibody and magnetic Protein A beads was carried out following co-transfection into A375 cells of wild-type non-tagged ERCC1 with human ubiquitin wild-type and ubiquitin (7R) (Figure 1E). 5 mM iodoacetamide was added to all the buffers just before use to inhibit deubiquitinase activity. The full gel of the input samples (Supplementary Figure 2C) shows that a ubiquitination ladder can also be seen from overexpressed ERCC1 without immunopurification. In the IP panel, compared with the controls, two additional bands with sizes corresponding to ERCC1 modified by 3 or 4 ubiquitins were seen in the co-transfection with ubiquitin (WT). Importantly, the larger band was also present, but at lower intensity, in the co-transfection with ubiquitin (7R). The lower intensity may be because the mutations affect the ubiquitin structure, which could influence ubiquitin chain structure and the recognition sensitivity of the antibody. In both cases, the band was stronger following MG132 exposure, suggesting that that the stability of ERCC1 is indeed regulated by polyubiquitination involving Lys33 of ubiquitin and linear polyubiquitination.

### The XPF binding domain of ERCC1 is the key domain for proteasome-dependent degradation

**Figure 2:**
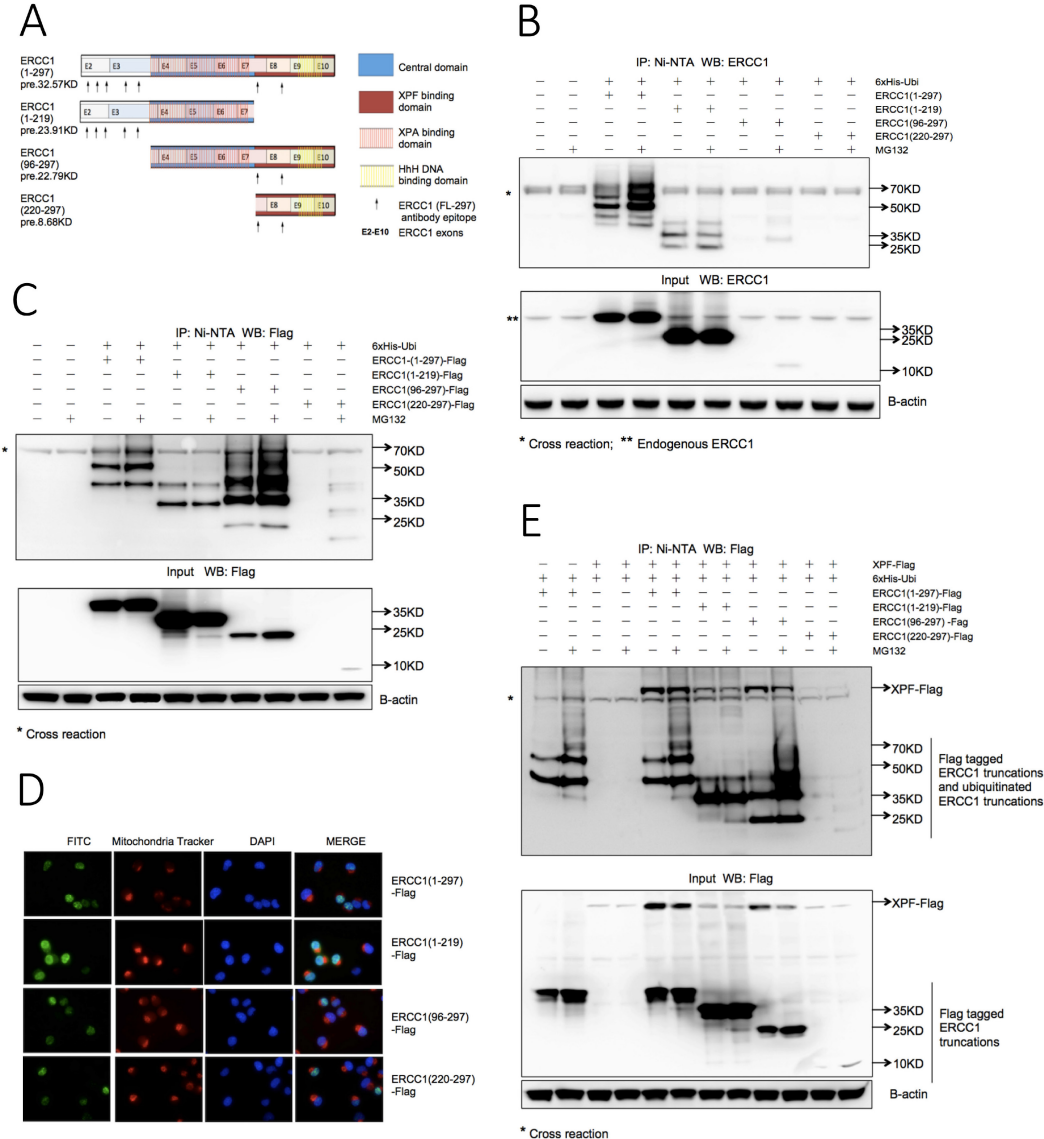
The XPF binding domain of ERCC1 determines the stability of both ERCC1 and XPF (**A**) Schematic of protein domains and exon boundaries for full-length (1-297) ERCC1 protein and deletion constructs (1-219), (96-297) and (220-297). Constructs were made with and without an N-terminal Flag tag. The predicted sizes (pre.) of non-tagged constructs are shown. Arrows beneath each construct indicate the location of epitopes for the ERCC1 polyclonal antibody (FL297). (**B**) Ubiquitination of ERCC1 deletion constructs. 6xHis-tagged wild-type ubiquitin was co-transfected into A375 cells in duplicate 60 mm dishes with non-tagged ERCC1 (1-297), (1-219), (96-297) and (220-297) for *in vivo* ubiquitination assay. After 24 h one dish from each pair was exposed to MG132 (25 μΜ) for 5 h. Material purified on the Ni beads was western blotted for ERCC1. A sample of the input material prior to purification was also blotted for ERCC1 and beta-actin. Note the less extensive ubiquitination ladder with lack of an MG132-induced increase in ubiquitination with ERCC1 (1-219) compared to the (1-297) construct and the difficulty of detecting the (220-297) and (96-297) constructs. (**C**) Ubiquitination of Flag-tagged ERCC1 deletion constructs. The *in vivo* ubiquitination assay shown in Panel B was repeated using Flag-tagged ERCC1 constructs and an antibody against the Flag tag. Note again the less extensive ubiquitination ladder and lack of an MG132-induced increase in ubiquitination with ERCC1 (1-219) compared to all the other constructs, the particularly strong ubiquitination ladder with the (96-297) construct and the reduced levels of non-modified ERCC1 with both (96-297) and (220-297) constructs. (**D**) Nuclear location of Flag-tagged ERCC1 deletion constructs. 1×10^5^ A375 cells growing on coverslips in 6-well plates were transfected with 1 μg of each Flag-tagged ERCC1 truncation. 24 h later live cells were stained with mitochondria tracker to identify cytoplasm before cells were fixed for immunofluorescence with an FITC-conjugated anti-Flag antibody and DAPI staining to identify nuclei. Images in the three individual fluorescence channels and an image merge are shown. Note the predominantly nuclear location of proteins encoded by all ERCC1 constructs. (**E**) Ubiquitination of ERCC1 is not influenced by cotransfection of XPF, but XPF depends on ERCC1 for stability. Flag-tagged ERCC1 (1-297), (1-219), and (96-297) constructs were co-transfected into A375 cells with 6xHis-tagged ubiquitin and Flag-tagged full-length XPF in duplicate dishes. Co-transfections of Flag-tagged ERCC1 (1-297) with 6xHis-tagged ubiquitin and of Flag-tagged XPF and 6xHis-tagged ubiquitin served as controls. After 24 h one dish from each pair was exposed to MG132 (25 μΜ) for 5 h and the *in vivo* ubiquitination assay was performed as described. The positions of Flag-tagged ERCC1 truncations and ubiquitinated truncations are indicated on the gels. Note that the amount of XPF is dependent on the presence and stability of the ERCC1 (220-297) domain.

We next investigated the key domain(s) on ERCC1 for ubiquitination-dependent degradation. The domain structure of the ERCC1 protein is shown in Figure 2A. Since the epitopes for our favoured ERCC1 polyclonal antibody (FL297) were not evenly distributed between the domains, we made deletion constructs with and without an N-terminal Flag tag: ERCC1 (1-297), (1-219), (96-297) and (220-297) (Figure 2A). Constructs were co-transfected into A375 cells with 6xHis-tagged wild-type ubiquitin and the ubiquitination assay was performed. For the non-tagged samples (Figure 2B), the full-length ERCC1 (1-297) panel showed the same ubiquitination ladder, at increased intensity following MG132 exposure, as seen in Figure 1. The ERCC1 (1-219) input samples showed much stronger signals than for ERCC1 (1-297), but with no increase after MG132 exposure. In the ERCC1 (1-219) IP samples, there was no extensive ubiquitination ladder and again there was no effect of MG132 exposure. We conclude that ERCC1 (1-219) is not required for ubiquitin-dependent proteasome degradation. Only weak bands were present in the MG132-exposed ERCC1 (96-297) samples and no bands were detected for ERCC1 (220-297). This could be as a result of the lack of sufficient epitopes for detection by the ERCC1 antibody, or increased protein degradation.

This uncertainty was resolved when the assay was repeated with the Flag-tagged ERCC1 constructs and an anti-Flag antibody (Figure 2C). For ERCC1 (1-297)-Flag and (1-219)-Flag the result with the non-tagged constructs was confirmed. As expected, the ERCC1 (96-297)-Flag and ERCC1 (220-297)-Flag panels showed stronger signals than the non-tagged panels. For ERCC1 (96-297)-Flag, the ERCC1 band in the input samples was much weaker than with ERCC1 (1-297)-Flag and (1-219)-Flag, with an increase after MG132 exposure. Intriguingly, the ubiquitination ladder for the ERCC1 (96-297)-Flag IP sample was much stronger than for ERCC1 (1-297)-Flag and (1-219)-Flag, particularly after MG132 exposure. This indicates that the presence of the N-terminal (1-95) domain can partially protect ERCC1 from proteasome degradation. For ERCC1 (220-297)-Flag input and IP samples, only the MG132-exposed samples showed detectable bands, indicating that expression of this domain alone results in rapid degradation by the ubiquitin-dependent proteasome pathway.

To exclude the possibility that ERCC1 deletion constructs were interacting differently with the ubiquitin-dependent proteasome degradation pathway due to the altered subcellular location of their encoded ERCC1 proteins, immunofluorescence was carried out for the Flag-tagged ERCC1 constructs in A375 cells, together with nuclear and cytoplasmic markers (Figure 2D). As expected, all ERCC1 proteins were predominantly located in nuclei, allowing us to conclude that the key residue(s) involved in ubiquitin-dependent proteasome degradation are indeed present in the XPF binding domain of ERCC1, residues 220-297.

### XPF is not ubiquitinated but its stability is affected by ERCC1 ubiquitination

Could the ubiquitin-dependent proteasome degradation of overexpressed ERCC1 simply be an artefact caused by lack of matching amounts of its XPF binding partner? To address this question, we repeated the ubiquitination assay on the Flag-tagged ERCC1 truncations after co-transfecting a Flag-tagged full-length XPF construct (Figure 2E). The input samples and characteristic ubiquitination patterns of full-length and truncated ERCC1 proteins were all unaffected by the presence of overexpressed XPF. There was no evidence for XPF ubiquitination in any samples, indicating that it is not itself a substrate for the ubiquitin-dependent proteasome degradation pathway. However, presence of Flag-tagged XPF was dependent on overexpression of ERCC1. In both IP and input samples the XPF signal was very weak or undetectable in the no ERCC1 overexpressed samples, the co-transfection with ERCC1 (1-219)-Flag, which is without an XPF binding domain, and the co-transfection with ERCC1 (220-297)-Flag where the ERCC1 is rapidly degraded. Moreover, for XPF co-transfected with ERCC1 (1-297)-Flag and ERCC1 (96-297)-Flag, XPF was reduced in both the MG132-exposed input and IP samples whereas full-length ERCC1 or ERCC1 (96-297) was accumulated. We conclude that the ubiquitin-dependent proteasome degradation of ERCC1 is not due to lack of its XPF binding partner. However, XPF is dependent on the presence of ERCC1 for its stability and its stability is reduced by ubiquitination of ERCC1.

### The C-terminal (HhH)_2_ XPF binding domain of ERCC1 is polyubiquitinated in a non-conventional lysine-independent manner

We next investigated the ubiquitination site(s) in the C-terminal (HhH)_2_ XPF binding domain of ERCC1 necessary for proteasome degradation. While serine hydroxyl and cysteine thiol groups of the substrate can be modified by ubiquitin [37–39], ubiquitin conjugation is mostly through an amide isopeptide bond between the C-terminus of ubiquitin and a ε-amino group of a lysine residue on the target protein [40]. Three of the five lysines present, Lys243, Lys281 and Lys295, were reported to be modified in a global mass spectrometry assay (phosphosite plus, http://www.phosphosite.org/homeAction.do). The crystal structure of the strong interaction between the double helix-hairpin-helix (HhH)_2_ domains of ERCC1 and XPF has been determined [20]. We found that the ε-amino groups of all five ERCC1 lysines were exposed in this structure, so having the potential to be ubiquitinated (Supplementary Figure 3A). Of the five hydrogen bond pairs between ERCC1 and XPF (Gly258-Phe894, Arg234-Lys843, Glu261-Ile890, Leu289-Ala863 and Phe293-Lys860) all, apart from Glu261-Ile890, were linked in hydrogen bond chains with ERCC1 lysines (Supplementary Figure 3B and 3C). In addition, the ring of ERCC1 Phe293 fits perfectly into a hydrophobic cavity formed by the (HhH)_2_ motif of XPF. ERCC1 lacking the last four residues including Lys295 still binds to XPF, whereas deletion of the next residue, Phe293, eliminates XPF binding [16]. Thus, there is the potential for ubiquitination of any of these ERCC1 lysines to destabilise the binding of ERCC1 to XPF.

**Figure 3:**
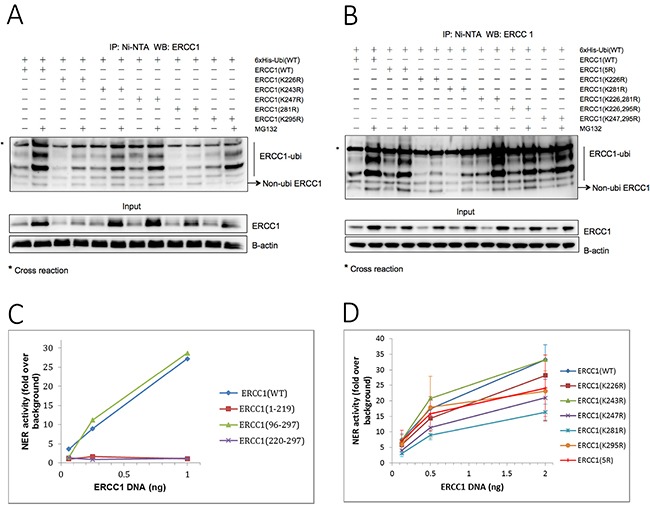
ERCC1 is ubiquitinated in a non-conventional lysine-independent manner (**A**) 6xHis-tagged ubiquitin was co-transfected into A375 cells with individual point mutants in the XPF-binding domain of ERCC1, where each of the five lysines was mutated to arginine, ERCC1 (K226R), (K243R), (K247R), (K281R) and (K295R). Wild-type ERCC1 was used as positive control. Each transfection was carried out in duplicate. After 24 h, one dish from each pair was exposed to MG132 (25 μΜ) for 5 h and then the *in vivo* ubiquitination assay was performed. Material purified on Ni beads was western blotted for ERCC1. A sample of the input material prior to purification was also blotted for ERCC1 and B-actin. The positions of the ubiquitinated ERCC1 ladder and of non-ubiquitinated ERCC1 are indicated. Note the reduced levels of ERCC1 ubiquitination and of total ERCC1 in the MG132-exposed K226R and K281R mutants. (**B**) *In vivo* ubiquitin assay with point mutants ERCC1 (K226R) and (K281R), lysine combination mutants in the XPF binding domain, ERCC1 (K226, 295R) and (K247, 295R), and ERCC1 (5R), where all 5 Lysines were mutated to Arginine. Note again the reduced levels of ERCC1 ubiquitination and of total ERCC1 in the MG132-exposed K226R and K281R mutants, but that ubiquitination in the double mutant ERCC1 (K226, 281R) was not reduced. Ubiquitination was also not reduced in the ERCC1 (5R) multiple mutant. (**C**) Validation of a nucleotide excision repair (NER) assay for mutant ERCC1 constructs. ERCC1-null A375 melanoma cells plated into 96-well plates were co-transfected 24 h later with ERCC1 wild-type or deletion constructs, (1-219), (96-297) and (220-297), and a UV-damaged or non-damaged GFP plasmid with a control luciferase plasmid and then assayed for GFP and luciferase activity after a further 48 hr. NER activity is plotted as fold over background in non-transfected cells against amount of ERCC1 plasmid used in each transfection. Note the complete lack of NER activity with ERCC1 (1-219) and ERCC1 (220-297) plasmids. (**D**) Lysines in the XPF binding domain of ERCC1 are not required for NER activity. NER assay with ERCC1 (WT) and point mutants (K226R), (K243R), (K247R), (K281R), (K295R) and multiple mutant (5R) in ERCC1-null A375 melanoma cells. Note that none of the ERCC1 mutants tested had significantly lower levels of NER activity than the wild-type construct.

Ubiquitination assays were carried out as before with ERCC1 constructs where lysines in the XPF binding domain of ERCC1, (K226R), (K243R), (K247R), (K281R) and (K295R), were individually mutated to arginine. Mutants were co-transfected into A375 cells with 6xHis-tagged wild-type ubiquitin (Figure 3A). Compared with the ERCC1 wild-type samples, the ERCC1 input protein level and ubiquitination ladder were reduced in both ERCC1 (K226R) and ERCC1 (K281R) samples. We cannot conclude just from this result that these are key lysines for ubiquitination because these mutations might affect the ERCC1 structure and so alter the recognition by E3 ligase during ERCC1 ubiquitination. To investigate further, we repeated the assay with combination mutants ERCC1 K(226, 281)R, K(226, 295)R and K(247, 295)R, where two lysines were mutated to arginine and ERCC1 (5R), where all five lysines were mutated (Figure 3B). None of the combination mutants prevented ERCC1 ubiquitination, indeed the ERCC1 K(226, 281)R samples showed a stronger ubiquitination ladder than the wild type. We conclude that the proteasome-dependent degradation of ERCC1 relies on non-conventional ubiquitin modification of the XPF binding domain that is independent of lysine residues.

### The C-terminal (HhH)_2_ XPF binding domain of ERCC1 is essential for nucleotide excision repair but the constituent lysine residues are not required

Although none of the lysines in the XPF binding domain of ERCC1 were essential for ERCC1 polyubiquitination, this did not preclude a role in NER activity. A transfection-based NER assay originally developed for use in ERCC1-proficient A375 cells to assess the efficacy of small molecule ERCC1 inhibitors [27] was first validated for use with ERCC1 constructs in ERCC1-null A375 cells that were generated by CRISPR/Cas9 genome editing specifically for this purpose. ERCC1-null A375 cells were co-transfected in 96-well plates with ERCC1 wild-type and deletion mutants ERCC1 (1-219), (96-297) and (220-297), together with an XPF expression plasmid, a UV-damaged or non-damaged GFP plasmid and a transfection control luciferase plasmid. GFP fluorescence and luciferase luminescence were measured 48 h after transfection. DNA repair activity of the deletion mutant ERCC1 (96-297) was equivalent to the wild-type ERCC1, but deletion mutants ERCC1 (1-219) and (220-297) showed no DNA repair activity at all (Figure 3C). This assay demonstrated the essential roles of the central and XPF binding domains of ERCC1 and so validated it to investigate the effect on NER of the ERCC1 point mutants.

All five of the single lysine mutants tested, ERCC1 (K226R), (K243R), (K247R), (K281R), (K295R), and ERCC1 (5R), where all five lysines were mutated, showed good NER activity compared to the deletion mutants ERCC1 (1-219) and (220-297) (Figure 3D). Although all except (K243R) showed lower activity than the wild-type control, in no case were any of the differences significant and we conclude that these lysine residues are not involved in the function and stability of ERCC1-XPF.

### Homodimerization of over-expressed ERCC1 occurs and ERCC1 (220-297) can be used to destabilise co-transfected wild-type ERCC1

Our observation, that over-expressed ERCC1 was stable in the absence of matching levels of XPF, led us to hypothesise that, contrary to conventional understanding, this stability could result from ERCC1 homodimer formation. Theoretical support comes from the very high similarity at secondary and tertiary structural levels of the (HhH)_2_ folds of ERCC1 and XPF in the crystal structure of the ERCC1-XPF heterodimer [20], see Supplementary Figure 4A. In addition, archaeal XPF homodimers also interact via their central nuclease domains, and the crystal structure of the central domain of ERCC1 [19] has a strikingly similar fold to the nuclease domain of the archaeal Mus81/XPF homologue [41], see Supplementary Figure 4B.

**Figure 4:**
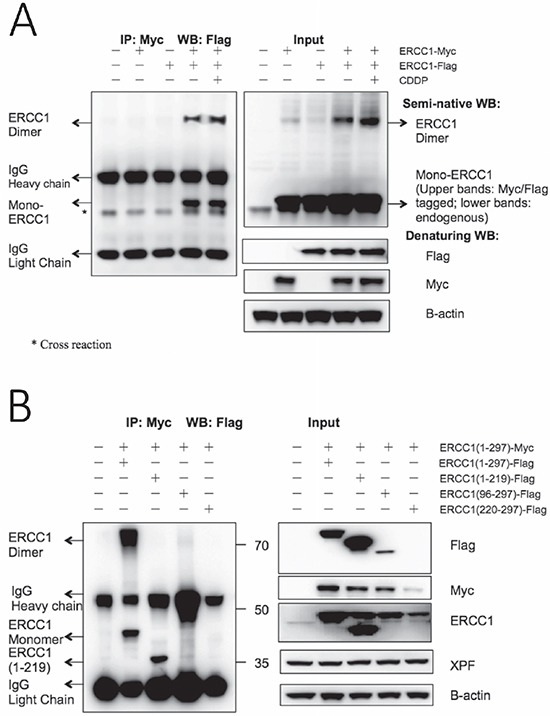
Homodimerization of overexpressed ERCC1 occurs and construct ERCC1 (220-297) can dramatically destabilise co-transfected wild-type ERCC1 (**A**) Flag-tagged wild-type ERCC1 was co-transfected with Myc-tagged wild-type ERCC1 in duplicate dishes of A375 cells. Non-transfected, ERCC1-Myc-only and ERCC1-Flag-only transfections were used as controls. After 24 h one of the co-transfected dishes was exposed to 25 μM cisplatin (CDDP) for 5 h. Immunoprecipitation was then performed with an antibody to the Myc tag linked to magnetic beads. Western blotting was performed for the IP samples with anti-Flag antibody on a conventional denaturing gel. To visualise intact ERCC1 homodimers, a semi-native western blot was run with non-reduced and non-boiled input samples and blotted with an anti-ERCC1 antibody. Blots of input samples, run on conventional denaturing gels, with anti-Flag, anti-Myc and anti-B-actin antibodies are also shown. The positions of ERCC1-Flag monomers and homodimers, IgG heavy and light chains and a cross reacting band are all indicated. Note: ERCC1-Flag monomers and homodimers are present in the co-transfected IP samples, but not in the ERCC1-Flag only transfection control, and ERCC1 homodimers are also present on the blot of input samples on the semi-native gel. (**B**) Myc-tagged wild-type ERCC1(1-297) was co-transfected with Flag-tagged wild-type ERCC1(1-297) and Flag-tagged truncations (1-219), (96-297) and (220-297). 24 h after transfection, immunoprecipitation was performed with an antibody to the Myc tag linked to magnetic beads and western blotting was performed for the IP samples as before with anti-Flag antibody. Mol. wt. markers (kDa) are shown. Blots of input samples run on conventional denaturing gels with anti-Flag, anti-Myc and anti-B-actin antibodies are also shown. The positions of ERCC1(1-297)-Flag monomers and homodimers, ERCC1(1-219)-Flag monomers and of IgG heavy and light chains are indicated. Note that ERCC1-Flag monomers and homodimers are again present in the ERCC1(1-297)-Flag and ERCC1(1-297)-Myc co-transfected IP sample, that ERCC1(1-219)-Flag was also pulled down by ERCC1(1-297)-Myc and that the level of Myc-tagged wild-type ERCC1 was greatly reduced in the co-transfection with ERCC1 (220-297)-Flag.

We investigated this hypothesis following the co-transfection of separate Flag- and Myc-tagged ERCC1 constructs into A375 cells by immunoprecipitation with a magnetic bead-linked antibody to the Myc tag (Figure 4A). Non-transfected, Flag-tagged ERCC1 transfected-only and Myc-tagged ERCC1 transfected-only samples were used as controls. An additional co-transfection was set up to investigate the possibility that ERCC1 homodimerization may be affected by cisplatin treatment. Conventional western blotting was performed as before, with Flag-tag antibody for the IP samples and Myc-tag antibody and Flag-tag antibody, together with B-actin antibody as the internal control, for the input samples. Flag-tagged ERCC1 was only seen after IP if co-transfected with Myc-tagged ERCC1, showing that Flag-tagged ERCC1 was pulled down by the Myc-tagged ERCC1. Moreover, there was a band detected by the Flag-tag antibody at double the normal size of ERCC1 and the intensity of this band was increased after cisplatin treatment. In addition to this conventional western blot with the IP samples (SDS PAGE gel with boiled samples under reducing conditions), we ran a semi-native western blot (SDS PAGE gel, non-boiled and non-reduced samples) with the input samples using the ERCC1 antibody. In addition to endogenous ERCC1 and overexpressed Flag- and Myc-tagged ERCC1 at the expected size for monomers, a band at double the size of ERCC1 was present in all ERCC1- transfected samples and this band was also increased in intensity after cisplatin treatment. We conclude that homodimerization of over-expressed ERCC1 does indeed occur.

A further immunoprecipitation was carried out to investigate if ERCC1 homodimerization occurs through both (HhH)_2_ and central domains (Figure 4B). Full length Myc-tagged ERCC1 was co-transfected with Flag-tagged full-length ERCC1 or truncations (1-219, 96-297 and 220-297) and immunoprecipitation was performed as before, with conventional western blots. For the IP samples, the Flag-tagged full-length ERCC1 monomer was pulled down by the Myc-tagged ERCC1 as before, together with a band of ERCC1 homodimer size, so confirming the previous result. Intriguingly, the Flag-tagged ERCC1 (1-219) truncation lacking the (HhH)_2_ domain was also pulled down by Myc-tagged ERCC1, but with no band corresponding to the size of mixed ERCC1 homodimer. Truncations of Flag tagged ERCC1 (96-297) and ERCC1 (220-297) were not pulled down and the results with the input samples again indicated that these truncations were much more unstable than the other ERCC1 constructs. These results indicate that homodimerization of ERCC1 is likely through the central as well as the (HhH)_2_ domains, but that the central domain interaction is weaker. Moreover, in the input results panel, the level of full-length Myc-tagged ERCC1 was clearly reduced by co-transfection with Flag-tagged ERCC1 truncations, particularly the unstable ERCC1 (220-297).

### Homodimerization of endogenous ERCC1

To investigate possible homodimerization of ERCC1 at endogenous expression levels, size exclusion chromatography was performed on native lysates from non-transfected control and cisplatin-treated A375 melanoma cells. A semi-native blot was also performed to identify potential ERCC1 homodimers as well as the normal denaturing western blot. In addition to antibodies against ERCC1 and XPF, an anti-XPA antibody was used as a marker for NER complexes and an anti-EME1 antibody was used for interstrand crosslink (ICL) repair complexes (Supplementary Figure 5). For the non-treated sample, the normal western blot (Supplementary Figure 5Aa) showed that the distribution of ERCC1 and XPF was very similar, with both peaking in fractions from 158-440 kD. This coincided with a small XPA peak, indicating that these are NER complexes. ERCC1 and XPF were also present in higher molecular weight fractions extending beyond the largest marker at 689 kD, which was the peak for the ICL complex marker protein EME1, indicating that ERCC1 and XPF in these fractions are engaged in ICL repair, rather than in NER. ERCC1, but not XPF monomers were also present in smaller fractions extending down to 75 kD. The semi-native western blot (Supplementary Figure 5Ab) was very similar but, intriguingly, there was also a band at ERCC1 homodimer size in two of the same fractions of around 80 kD as the ERCC1 monomer sub-peak. For the cisplatin-treated sample, the normal western blot (Supplementary Figure 5Ba) showed more distinct peaks for ERCC1 and XPF in ICL repair complexes, in addition to the NER complex peaks. In the semi-native western blot (Supplementary Figure 5Bb), bands of ERCC1 homodimer size were no longer present in fractions of around 80 kD, instead multiple ERCC1 bands were now present in NER fractions around 158 kD, including possible ERCC1-XPF heterodimers (ERCC1 33 kD, XPF 104 kD), and a band at around 80kD, the size of putative ERCC1 homodimers. We interpret these data as supporting the existence of ERCC1 homodimers at endogenous expression levels.

### Levels of endogenous ERCC1 and XPF are both reduced by expression of the ERCC1 (220-297) peptide

**Figure 5:**
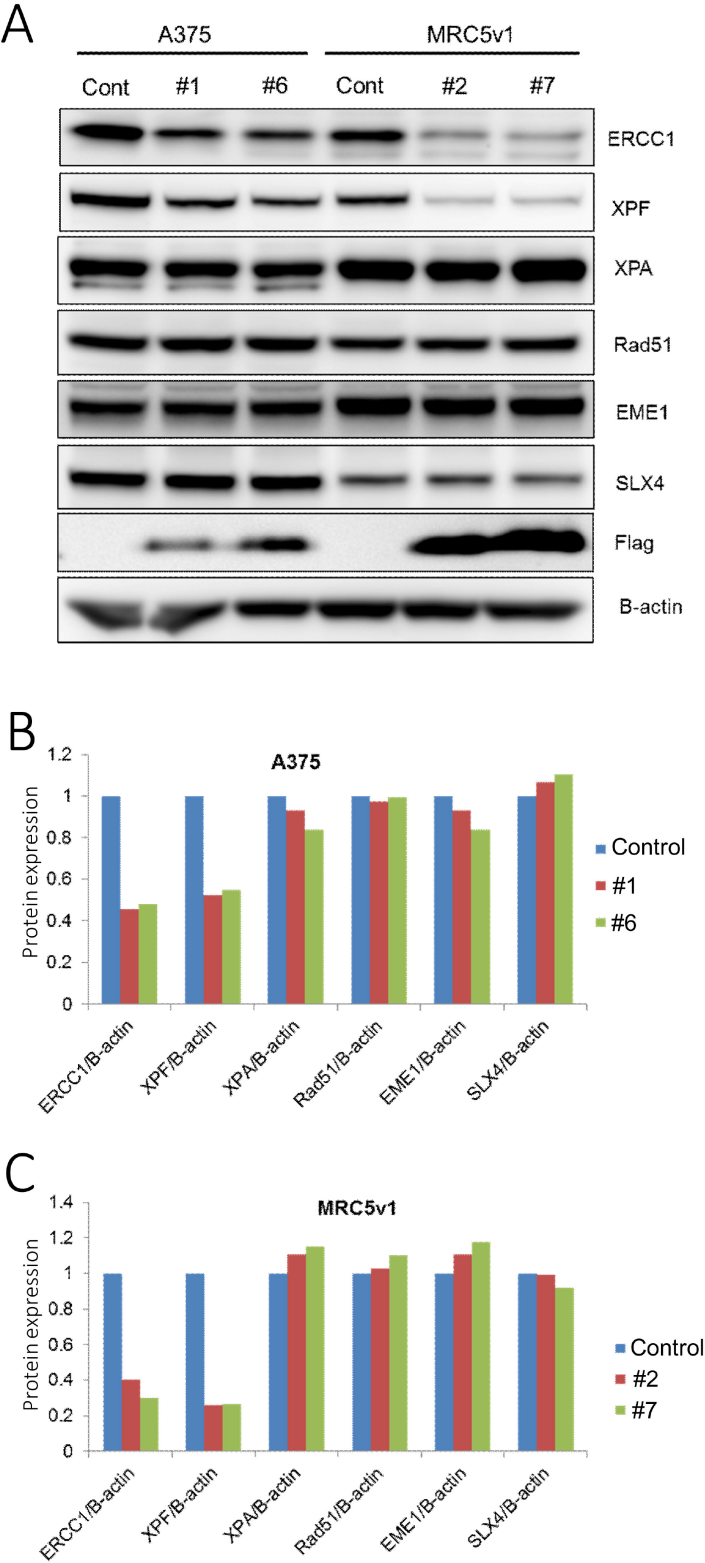
Levels of endogenous ERCC1 and XPF are both reduced by transfection with the ERCC1 (220-297) truncation A375 human melanoma cells and MRC5v1 immortalised human fibroblasts were transfected with the Flag-tagged ERCC1 (220-297) truncation, selection for the neo marker present on this plasmid was applied with G418 (2 mg/ml) and clones expressing the Flag-tag were identified. (**A**) Western blots of control A375 cells, A375 ERCC1 (220-297) expressing clones #1 and #6, control MRC5v1 cells and MRC5v1 ERCC1 (220-297) expressing clones #2 and #7. Blotting for ERCC1 (33 kDa), XPF (104 kDa), XPA (40 kDa, NER marker protein), RAD51 (37 kDa), EME1 (63 kDa) and SLX4 (200 kDa, all interstrand crosslink repair marker proteins), Flag tag (detecting Flag-tagged ERCC1 (220-297), 11.5 kDa) and beta-actin (42 kDa). (**B**) Histogram showing the level of each protein blotted in A375 ERCC1 (220-297) clones #1 and #6, expressed relative to B-actin and normalized to the level in A375 control cells. (**C**) Histogram showing the level of each protein blotted in MRC5v1 ERCC1 (220-297) clones #2 and #7, expressed relative to B-actin and normalized to the level in MRC5v1 control cells. Note the reduced levels of ERCC1 and XPF in all clones.

Having demonstrated that the ERCC1 (220-297) peptide can destabilise over-expressed levels of ERCC1 and XPF, we next investigated whether it was also able to reduce endogenous levels of both proteins and so inhibit NER and enhance the sensitivity of cancer cells to DNA damaging agents. A375 melanoma cells and MRC5v1 immortalised human fibroblasts were transfected with the Flag-tagged ERCC1 (220-297) truncation plasmid and G418 selection was applied for the neo marker on the plasmid. Resistant colonies were screened for expression of the Flag tag and two Flag tagged- ERCC1 (220-297) expressing clones of each cell type were analysed by western blotting (Figure 5 and Supplementary Figure 6). Levels of ERCC1 and XPF were reduced by more than 50% in both A375 clones analysed compared to control A375 cells, while levels of another NER protein (XPA) and three ICL repair proteins (RAD51, EME1 and SLX4) were unaffected (Figure 5A and 5B). ERCC1 and XPF were also reduced by more than 60% in both MRC5v1 clones (Figure 5A and 5C).

### The ERCC1 (220-297) peptide inhibits nucleotide excision repair and increases sensitivity to DNA damaging agents

**Figure 6:**
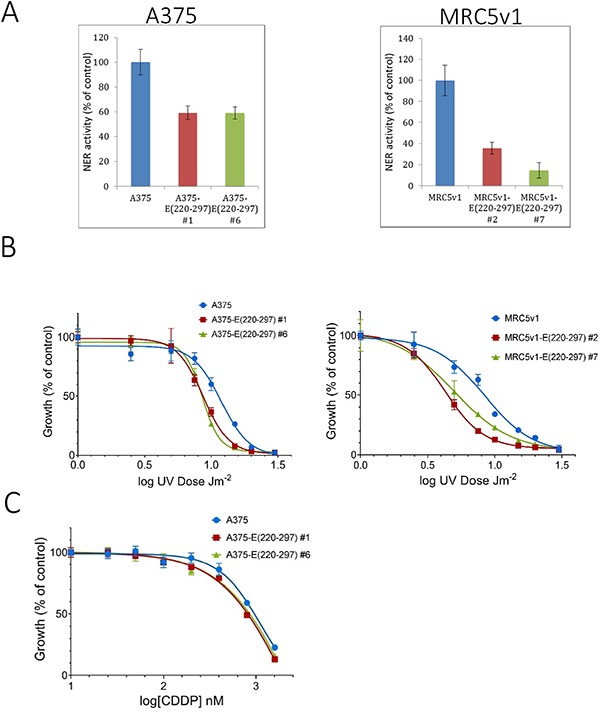
Expression of the ERCC1 (220-297) peptide inhibits nucleotide excision repair and sensitises cells to UV-induced DNA damage and cisplatin (**A**) Inhibition of NER. Cells from A375, A375 ERCC1 (220-297) clones #1 and #6, MRC5v1 and MRC5v1 ERCC1 (220-297) clones #2 and #7 were plated in 96-well plates, co-transfected 24 hr later with UV-damaged or non-damaged GFP and control luciferase plasmids and assayed for GFP and luciferase activity after a further 24 hr. Values plotted are the mean NER activity (±SEM) from three independent experiments as a percentage of activity in normal control cells. (**B**) Increased sensitivity to UV-induced DNA damage. 10^4^ cells from A375, A375 ERCC1 (220-297) clones #1 and #6, MRC5v1 and MRC5v1 ERCC1 (220-297) clones #2 and #7 were plated in duplicate 35 mm dishes. 24 h later dishes were exposed to 1-20 Jm^-2^ UVC. 5 days later dishes were fixed and stained with Sulphorhodamine B [53]. For each cell line, growth is expressed as the percentage of the non-irradiated control. Values plotted are mean % growth (± SEM) from two independent experiments. (**C**) Increased sensitivity to cisplatin. A standard 5-day SRB growth assay in 96-well plates was carried out. 500 cells/well from A375, A375 ERCC1 (220-297) clones #1 and #6 were plated in quadruplicates and exposed to 25-1600 nM cisplatin (CDDP) for five days before plates were fixed and stained. For each cell line, growth is expressed as the percentage of the non-cisplatin-treated control. Values plotted are mean % growth (± SEM) from two independent experiments.

The NER assay was carried out, as described in Materials and Methods, on the same two Flag-tagged ERCC1 (220-297) -expressing clones of A375 and MRC5v1 as above. Cells in 96-well plates were co-transfected with UV-damaged or non-damaged GFP plasmids and control luciferase plasmids. 24 hr later, the GFP and luciferase activity were measured and NER activity was calculated (Figure 6A). Compared with the control cells, NER activity of both A375 ERCC1 (220-297) clones was reduced by 40%, while the reduction for the two MRC5v1 clones was greater at 65% and 85%. These reductions in NER activity relative to the cell type control were all highly significant (*p* < 0.025 by Student's *t* test).

To determine whether these large ERCC1 (220-297) peptide-induced reductions in NER activity resulted in increased sensitivity to DNA damaging agents, we carried out standard 5-day growth assays following UVC irradiation (Figure 6B). We chose this agent initially because UVC-induced DNA damage is repaired exclusively by NER. The IC50 for A375 cells was 11.4 Jm^-2^ (95% confidence interval 10.9-11.9 Jm^-2^), while IC50s for A375-E (220-297) clones #1, 8.7 (95% confidence interval 8.4-9.0), and #6, 8.5 (95% confidence interval 8.4-8.7) Jm^-2^ were 25% lower. The IC50 for MRC5v1 cells was 8.3 (7.9-8.8) Jm^-2^, while the IC50 for MRC5v1-E (220-297) clone #2 was 45% lower at 4.5 Jm^-2^ (95% CI 4.4-4.6) and the IC50 for clone #7 was 35% lower at 5.3 Jm^-2^ (95% CI 5.0-5.6). These increases in UV sensitivity relative to the cell type controls were all significant (*p* < 0.05). The effect of the ERCC1 (220-297) peptide-induced reduction in levels of ERCC1-XPF on sensitivity to the chemotherapeutic cisplatin was less pronounced. The IC50 value for A375-E (220-297) clone #1 (0.81 μM) was 15% less than the A375 control (0.96 μM), IC50 for clone #6 (0.86 μM) was 10% lower, but these differences were not significant (*p* > 0.05) (Figure 6C). The MRC5v1-E (220-297) clones showed no obvious increased sensitivity compared to control MRC5v1 cells (data not shown). Lower levels of ERCC1-XPF activity are known to be sufficient for the repair of cisplatin-induced ICLs than are required for NER [42]. Nevertheless, we conclude that the ERCC1 (220-297) peptide causes major reductions in NER activity and increased sensitivity to DNA damaging agents.

## DISCUSSION

Chemotherapy with DNA damaging agents remains the mainstay of current therapy for the majority of common cancers. The structure-specific endonuclease ERCC1-XPF is involved in the repair of DNA damage caused by many of these agents and there has been much interest in the use of ERCC1-XPF as a predictive marker of response to chemotherapy (reviewed in [4]). We validated ERCC1 as a therapeutic target by showing that a genetically engineered ERCC1-deficient mouse model of melanoma was uniquely sensitive to the chemotherapeutic cisplatin [23]. The ERCC1-deficient melanoma cells used were 10-fold more sensitive to cisplatin, leading us to conclude that biochemical inhibition of ERCC1-XPF would need to block > 90% of NER activity and achieve near 10-fold increased sensitivity to cisplatin to be of therapeutic value. As a guide, siRNA against ERCC1 or XPF resulted in only 2-3-fold increased cisplatin sensitivity [22]. Despite the availability of structural information and much endeavour we [27–29] and others [24–26] had been unable to identify ERCC1-XPF inhibitors of sufficient potency and specificity in preclinical models to justify further development.

Initial *in silico* screening identified a compound binding to the XPA-interacting pocket of ERCC1 that enhanced the UV sensitivity of human colon cancer cells by 2-fold, but had a weaker effect on cisplatin sensitivity [24, 25]. Our best *in silico* screening-derived inhibitor of the interaction between ERCC1 and XPF was active in an NER assay in melanoma cells (IC50 20 μM), but caused only a small (1.3-fold) reduction in the cisplatin IC50 [27]. While an independently derived ERCC1-XPF interaction inhibitor caused modest enhanced sensitivity to cisplatin in two cancer cell lines and was apparently able, at high concentrations (up to 500 μM), to disrupt the ERCC1-XPF interaction in cell extracts [26]. We had more success using high throughput screening to identify XPF endonuclease inhibitors with specificity for ERCC1-XPF over related endonucleases [27]. The two best inhibitors gave strong inhibition of NER in melanoma cells (IC50 < 10 μM), but again only increased the cisplatin sensitivity by up to 2-fold. While extensive medicinal chemistry on the two compounds was able to identify derivatives with improved activity against ERCC1-XPF endonuclease activity in an *in vitro* assay (IC50 < 1 μM), with > 10-fold selectivity for ERCC1-XPF over the related FEN-1 endonuclease, there was no improvement in the 2-fold increased sensitivity to cisplatin [28, 29].

The investigation of post-translational modification and stability reported here led us to an alternative approach to target ERCC1-XPF. However, while this work was in progress, more encouraging results for conventional ERCC1-XPF inhibitors have been reported [43, 44]. In particular, an ERCC1-XPF endonuclease inhibitor (IC50 < 1 μM), with specificity against another endonuclease and, despite only showing ˜2-fold enhanced sensitivity to cisplatin, potentiated cisplatin activity in a lung cancer xenograft model [44].

We found that the stability of ERCC1, but not of XPF, was regulated by ubiquitination. The consistent accumulation of ERCC1, but not XPF, after proteasome inhibitor MG132 exposure strongly indicated the involvement of the ubiquitin-dependent proteasome degradation pathway. Three ERCC1 lysine residues were previously found to be ubiquitinated in a global mass spectrometry assay (phosphosite plus, http://www.phosphosite.org/homeAction.do). Our ubiquitin assays focussed on the initial rungs of the ERCC1 ubiquitination ladder and we found that ERCC1 is likely modified by a chain formed by at least four ubiquitins. Although the mechanism of ERCC1 ubiquitination and the role this plays in the stability of ERCC1-XPF has not been studied previously, the USP45 protein has been found to bind to and deubiquitinate ERCC1. USP45 knockout cells showed reduced levels of NER and increased sensitivity to UV irradiation [45].

Polyubiquitination usually commences with an isopeptide linkage between the ubiquitin C-terminal carboxyl residue and the epsilon amino group of a Lys residue on the target. Additional ubiquitins are then added via any of the seven Lys residues (Lys6, Lys11, Lys27, Lys29, Lys33, Lys48 and Lys63), or the amino terminal Met residue [46]. Although polyubiquitination via Lys48 is most commonly involved in proteasome-dependent degradation of the target protein [47], our assays with ubiquitin Lys point and combination mutants and an antibody against linear ubiquitin chains indicated that, instead, both polyubiquitination via Lys33 and linear chain polyubiquitination of ERCC1 were occurring. The unexpected ubiquitination of ERCC1 observed with the human mutant ubiquitin construct, where all seven Lys residues were mutated, was also indicative of linear chain polyubiquitination [36]. These results indicate that an altered ERCC1 ubiquitination pattern can arise from two different types of alteration to ubiquitin. The first is mutation (Lys to Arg) of one of the Lys residues of ubiquitin that is involved in the ERCC1 ubiquitination process. Our data suggest that Lys33 is the only one of the seven Lys residues of ubiquitin that is involved in ERCC1 ubiquitination. The second is a structural alteration to ubiquitin resulting from alterations to one or more ubiquitin residues that affect the way that ubiquitin is processed by the ubiquitination machinery. Thus, the three amino acid difference between plant and human ubiquitin 7R results in a major difference in the ERCC1 ubiquitination pattern: a normal pattern with human 7R, but no ubiquitination with plant 7R. We suggest that, by extension, the Lys33 to Arg mutation within a normal human ubiquitin structure affects the ERCC1 ubiquitination pattern, but has no effect on the ubiquitination pattern in the altered 7R ubiquitin structure.

Using a series of non-tagged and Flag-tagged ERCC1 deletion constructs we showed that the XPF-binding domain of ERCC1, residues (220-297), was essential for ubiquitin-dependent proteasome degradation by increased intensity of the ubiquitination ladder following exposure to MG132 for all constructs containing this region and by the very low levels of protein detected in input samples from constructs containing the (220-297) domain only. ERCC1 residues (1-95) were not necessary for ubiquitin-dependent proteasome degradation, but instead helped to stabilise the protein. All ERCC1 proteins studied were predominantly located in nuclei, allowing us to exclude the possibility that the ERCC1 truncations were interacting differently with the ubiquitin-dependent proteasome degradation pathway due to an altered subcellular location.

By over-expressing XPF in addition to ERCC1 we showed that the ubiquitin-dependent proteasome degradation of over-expressed ERCC1 was not simply an artefact caused by lack of its XPF binding partner. There was no evidence that XPF was also a substrate for the ubiquitin-dependent proteasome degradation pathway. Instead, the presence of over-expressed XPF was dependent on over-expression of ERCC1 and its stability was reduced by ubiquitination of ERCC1. This could be an effective regulatory mechanism to prevent unwanted endonuclease activity (encoded by XPF) on non-damaged DNA.

Although examination of the crystal structure between the double helix-hairpin-helix (HhH)_2_ domains of ERCC1 and XPF [20] indicated the potential for polyubiquitination of any of the five Lys residues present in ERCC1 (220-297) to destabilise the binding of ERCC1 to XPF, none of the Lys point and combination mutants prevented ERCC1 polyubiquitination and we concluded that the proteasome-dependent degradation of ERCC1 involves non-conventional, lysine-independent ubiquitin modification of the XPF-binding domain. Serine hydroxyl and cysteine thiol groups of the substrate can also be modified by ubiquitin [37, 38]. Using a transfection-based NER assay [27] that we first validated for use in ERCC1-null A375 cells by demonstrating the essential roles of the central and XPF binding domains of ERCC1 for NER in the assay, we also found that none of the five lysines were essential for NER.

Our most unexpected finding was the clear demonstration that over-expressed ERCC1 can form homodimers. Heterodimerisation of ERCC1 with XPF had been considered essential for the stability of both proteins [16–18], although more recent siRNA experiments indicated that, while XPF protein levels were decreased when ERCC1 was knocked down, the converse was not true [22]. Our homodimer hypothesis arose when we found that over-expressed ERCC1 was stable in the absence of matching levels of XPF and was buoyed by the very high structural similarity between both the (HhH)_2_ and central domains of ERCC1 and XPF [19, 20, 41]. Following co-transfection of separate Flag- and Myc-tagged ERCC1 constructs and immunoprecipitation with a magnetic bead-linked antibody to the Myc tag, we found that Flag-tagged ERCC1 was specifically pulled down by the Myc-tagged ERCC1. Both Flag-tagged ERCC1 monomers and dimers were present on the denaturing western blot of the IP material and on the semi-native blot of input material, indicating an extremely strong homodimer interaction. Further immunoprecipitations between Flag-tagged ERCC1 truncations and full-length Myc-tagged ERCC1 showed that the central as well as the (HhH)_2_ domains were involved in homodimerisation, but that the central domain interaction was weaker, such that mixed Flag-tagged ERCC1 (1-219)/ Myc-tagged ERCC1 (1-297) homodimers could not be seen on the denaturing western blot.

Support for ERCC1 homodimerization also occurring at endogenous expression levels came from size exclusion chromatography. A semi-native western blot on native lysates from control A375 melanoma cells detected bands of ERCC1 homodimer size in two size fractions of around 80 kD. Following cisplatin treatment the putative ERCC1 homodimer bands had moved to higher molecular weight fractions of around 158 kD where NER complexes were located. The existence of ERCC1 homodimers at endogenous expression levels can also help to explain our initial result, where exposure to proteasome inhibitor MG132 resulted in accumulation of ERCC1 but not XPF. A substantial ERCC1 homodimer pool in addition to ERCC1-XPF heterodimers would be expected to lead to greater accumulation of ERCC1 than XPF after MG132 treatment, particularly if the ERCC1 homodimer pool also turned over more rapidly. This could also explain the result after cycloheximide exposure, where levels of ERCC1 dropped more rapidly than XPF.

We have provided structural information to support homodimerization occurring through the same strong hydrophobic and H-bonding interactions that operate to hold ERCC1-XPF heterodimers together. Whilst we have not determined the function of ERCC1 homodimerization, we consider that it could provide an alternative way to stabilise and store ERCC1 that serves to prevent unwanted non-repair-related endonuclease activity that resides with XPF in ERCC1-XPF heterodimers.

When the ERCC1 (220-297) peptide and XPF were overexpressed together (Figure 2E), there was a clear effect of the ERCC1 (220-297) peptide on reducing XPF levels. Whereas in the double-tagged ERCC1 homodimerization experiment, when the ERCC1 (220-297) peptide was overexpressed together with Myc-tagged ERCC1 wild-type (1-297) protein, but with endogenous levels of XPF (Figure 4B), there was a clear effect of the ERCC1 truncation on reducing levels of the overexpressed Myc-tagged ERCC1 wild-type protein, but why was there no observable effect on endogenous levels of XPF? We believe that overexpression of the target protein for destabilisation (XPF in the first instance, Myc-tagged ERCC1 wild-type protein in the second case) enlarges the detection window and so makes it easier to observe the destabilising effect of the ERCC1 (220-297) peptide in our transient transfection assays. We have shown that the ERCC1 (220-297) peptide is polyubiquitinated and rapidly degraded. Our results suggest that when newly synthesized ERCC1 (220-297) peptide dimerizes with either ERCC1 wild-type or XPF monomers via their C-terminal domains, the rapid polyubiquitination of the ERCC1 (220-297) monomer targets the resulting ERCC1 homodimers or ERCC1-XPF heterodimers for proteasome degradation.

These observations led us to investigate whether the ERCC1 (220-297) peptide could also destabilise endogenous ERCC1 and XPF in stably transfected cells. Levels of ERCC1 and XPF were indeed reduced by more than 50% in clones of A375 melanoma cells and MRC5v1 immortalised human fibroblasts expressing the Flag-tagged ERCC1 (220-297) truncation, while levels of other NER and ICL repair proteins were unaffected. This resulted in a 40% reduction in NER activity in both A375 ERCC1 (220-297) clones and a greater reduction (65 and 85%) in the two MRC5v1 ERCC1 (220-297) clones. In our NER assay the DNA repair activity of control A375 melanoma cells was four-fold higher than MRC5v1 fibroblast cells (data not shown). In addition to illustrating the importance of NER to this cancer type, it could also explain the greater ERCC1 (220-297) peptide-induced reduction in NER activity in MRC5v1 cells.

The ideal DNA damaging agent to show the effect of targeting ERCC1-XPF activity in cancer cells is short wave UVC irradiation which causes damage that is repaired exclusively by NER. The reduced levels of NER we induced did indeed lead to increased sensitivity to UVC-induced DNA damage. The IC50 values for the two A375-E (220-297) clones were 25% lower than for the A375 control, while IC50 values the MRC5v1-E (220-297) clones were 45% and 35% lower than for the MRC5v1 control. We also used a common chemotherapeutic, cisplatin, which causes multiple forms of DNA damage (monofunctional adducts and intrastrand crosslinks that are repaired by NER and interstrand crosslinks whose repair requires the involvement of a number of different repair pathways), in addition to also damaging proteins and other cellular components. Lower levels of ERCC1-XPF activity are known to be sufficient for the repair of cisplatin-induced interstrand crosslinks than are required for NER [42]. So the less pronounced effect of the ERCC1 (220-297) peptide-induced reduction in levels of ERCC1-XPF on sensitivity to cisplatin was not unexpected. Higher levels of the ERCC1 (220-297) peptide would be expected to drive the larger reductions in ERCC1-XPF levels needed to cause bigger increases in cisplatin sensitivity.

Although our ERCC1 ubiquitination studies were only carried out on human melanoma cells, we also demonstrated that the ability of the ERCC1 (220-297) peptide to destabilise both ERCC1 and XPF in human fibroblasts. This, coupled with the report that inactivating the USP45 protein that deubiquitinates ERCC1 in human myelogenous leukaemia and osteosarcoma cells leads to reduced levels of NER and increased sensitivity to UV irradiation [45], suggests that our therapeutic strategy is likely applicable to a wide range of human cancers. While delivering the 78-amino acid ERCC1 (220-297) peptide for therapeutic use would constitute a considerable challenge, recent developments with cell penetrating peptides suggest that the size in itself is not prohibitive and that such an approach merits consideration [48]. While this work was being carried out, another study [49] reported that expression of the XPF-interacting domain of ERCC1 in cancer cells resulted in increased sensitivity to DNA damaging agents, but without any investigation of the mechanism involved.

So, in the course of investigating the role of the proteasome-dependent protein degradation pathway in the stability of the key DNA repair protein ERCC1-XPF and the mechanism of its ubiquitination, we saw that XPF was dependent on ERCC1 for stability and made the surprising discovery that ERCC1 can also homodimerize in mammalian cells. This interaction, principally through the (HhH)_2_, but also involving the central domains of ERCC1, could be a mechanism to keep ERCC1 stable by preventing the ubiquitination of the key residue(s) in the (HhH)_2_ domain, without the risk of unwanted endonuclease activity from ERCC1-XPF heterodimers. We were able to exploit the instability of the ERCC1 (220-297) peptide to destabilise endogenous levels of both ERCC1 and XPF, resulting in major reductions in NER activity and increased sensitivity to DNA damaging agents. Given the importance of ERCC1-XPF as a target in cancer cells and the current lack of small molecule inhibitors with sufficient potency and specificity to enter clinical trials, if high levels of this peptide or a smaller mimic could be achieved in cancer cells, then it could represent a powerful new alternative therapeutic approach.

## MATERIALS AND METHODS

### Mammalian cells

Human A375 and C32 melanoma cells, authenticated by short tandem repeat profiling, were obtained from The European Collection of Cell Cultures (Salisbury, UK). An ERCC1 null derivative of A375 was generated using CRISPR/Cas9 genome editing and the loss of ERCC1 protein was confirmed by western blotting. The MRC5v1 cell line was kindly provided by Prof Alan Lehmann, Genome Damage and Stability Centre, University of Sussex. It was obtained by SV40 transformation of primary human fibroblasts [50]. All experiments were performed on cultures within 10 passages of their supply. Cells were maintained in DMEM medium (41965; Life Technologies Ltd., Paisley, UK), supplemented with 10% FCS, Non-Essential Amino Acids (11140-035; Life Technologies Ltd.), 1 mM Sodium Pyruvate, 2 mM L-glutamine and Penicillin (100 U/ml) –Streptomycin (100 mg/ml) at 37°C, 5% CO_2_.

### Plasmids

Full-length and truncated human ERCC1 constructs in mammalian expression vectors pcDNA3.1(-) /myc-His A (Life Technologies Ltd.) and p3xFLAG-CMV-14 (Sigma-Aldrich, Poole, UK) were constructed by standard cloning techniques. ERCC1 point mutants and combination mutants in pcDNA3.1(-)/myc-His A were generated using QuikChange^™^ Site-Directed (200518, Agilent Technologies UK Ltd., Edinburgh, UK) and Multi Site-Directed (200515, Agilent Technologies UK Ltd.) Mutagenesis Kits. Human 6x-His-Ubiquitin(WT) and plant 6x-His-Ubiquitin(7R) constructs in pcDNA3 (Life Technologies Ltd.) were supplied by Dr Lesley Stark (University of Edinburgh) [51]. Point and combination mutants of Human 6x-His-hUbiquitin(WT) were generated by site-directed mutagenesis as above. Human XPF (ERCC4) expressing plasmid pCMV6-ENTRY-ERCC4 (C-terminal Myc and DDK-tagged) was obtained from Cambridge Bioscience Ltd. (Cambridge, UK). The structures of all plasmids used were verified by DNA sequencing. See Supplementary Information for more details of constructs. Plasmids were transfected into A375 or MRC5v1 cells with Lipofectamine 2000, using conditions recommended by the supplier (Life Technologies Ltd).

### Western blotting

Protein extraction for denaturing SDS gels was carried out on ice using RIPA buffer (25 mM Tris-HCl pH 7.2, 150 mM NaCl, 1% Triton X-100, 1% deoxycholate, 1 mM EDTA, 20 mM NaF, 100 μM orthovanadate), with Roche complete protease inhibitor cocktail (Roche Products Ltd., Welwyn Garden City, UK) and western blotting was carried out as described [52]. Details of denaturing and semi-native gel systems and antibodies used are given in Supplementary Information.

### Ubiquitination assay

A375 cells expressing 6xHis-tagged ubiquitin and ERCC1 target constructs were lysed in 6 M guanidinium-HCl containing denaturing buffer and incubated overnight with Ni-NTA-agarose beads (30210, Qiagen Ltd., Manchester, UK). The beads were washed with 6 M guanidinium-HCl containing- and then 8 M urea-containing denaturing buffers. His-tagged ubiquitinated protein bound to the beads was eluted with 200 mM imidazole-containing buffer and western blotted. See Supplementary Information for full details.

### Immunocytochemistry, immunoprecipitation, size exclusion chromatography

See Supplementary Information for details of these assays.

### Nucleotide excision repair assays

Details and validation of the transfection-based assay to measure NER activity in A375 melanoma cells, involving UV-irradiated GFP and control luciferase plasmids, have been described [27]. Modifications of the assay to measure NER from ERCC1 constructs transfected into ERCC1-deficient A375 cells are described in Supplementary Information.

## SUPPLEMENTARY MATERIALS FIGURES AND TABLES



## Abbreviations

CDDP: cisplatin
GFP: green fluorescent protein
(HhH)_2_: paired helix-hairpin-helix
ICL: interstrand crosslink
NER: nucleotide excision repair
SRB: Sulphorhodamine B.
