# Supplementary material for: Disruption of DNA repair in cancer cells by ubiquitination of a destabilising dimerization domain of nucleotide excision repair protein ERCC1
Source: Oncotarget. 2017 Jul 21;8(33):55246–64. doi: 10.18632/oncotarget.19422 (PMC5589656; doi:10.18632/oncotarget.19422)
Supplement: Supplementary file 1 [file oncotarget-08-55246-s001.pdf]

Disruption of DNA repair in cancer cells by ubiquitination of a destabilising dimerization domain of nucleotide excision repair protein ERCC1

SUPPLEMENTARY MATERIALS

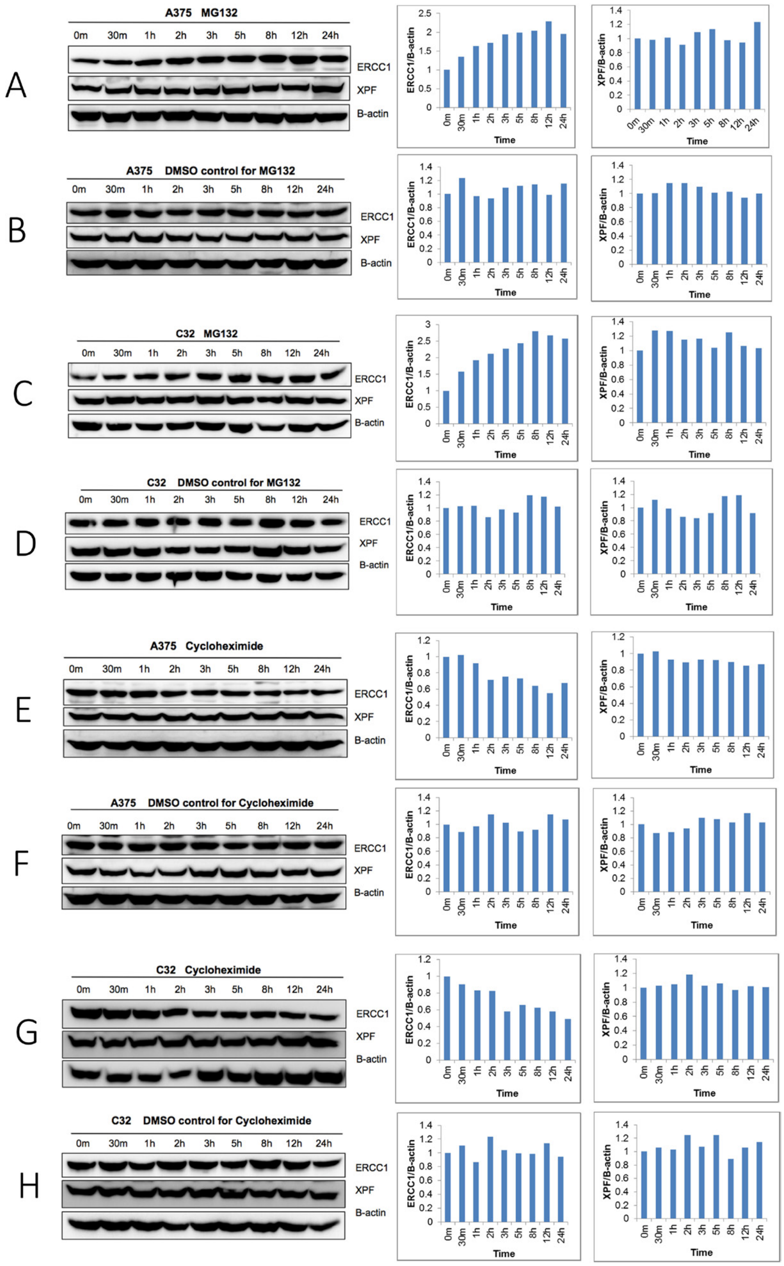

Supplementary Figure 1: ERCC1 but not XPF accumulates following MG132 exposure and decays following cycloheximide exposure. A375 and C32 human melanoma cells were plated in duplicate 60 mm dishes at densities sufficient to give 60-80% confluence at harvesting. One member of each duplicate was exposed to proteasome inhibitor MG132 (25 µM), or protein translation inhibitor cycloheximide (10 µg/ml) for times between 30 min and 24 h. Stock solutions of both drugs were made up in DMSO, so the other member of each duplicate was exposed to medium containing an equivalent concentration of DMSO as control. Protein extracts were prepared from each dish and western blotted. Histograms show the levels of ERCC1 and XPF relative to beta-actin, normalized to the non-exposed control. (**A**) A375, MG132. (**B**) A375, DMSO control for MG132. (**C**) C32, MG132. (**D**) C32, DMSO control for MG132. (**E**) A375, cycloheximide. (**F**) A375, DMSO control for cycloheximide. (**G**) C32, cycloheximide. (**H**) C32, DMSO control for cycloheximide.

A (Fig1A rerun combined input and IP samples)

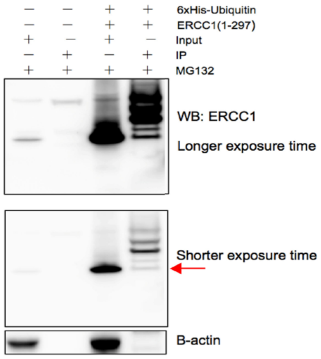

B (Fig1C IP full gel)

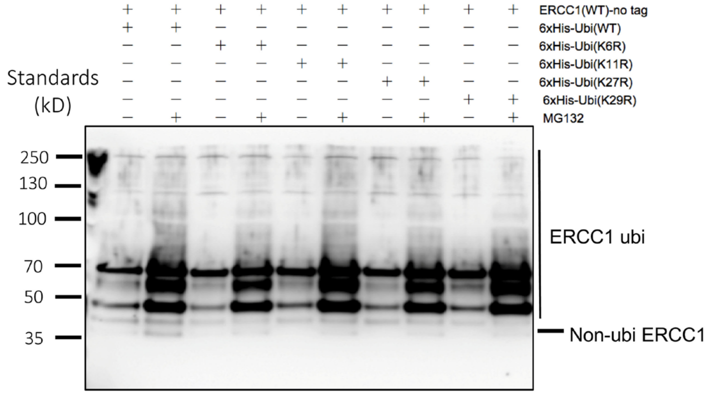

C (Fig1E Input full gel)

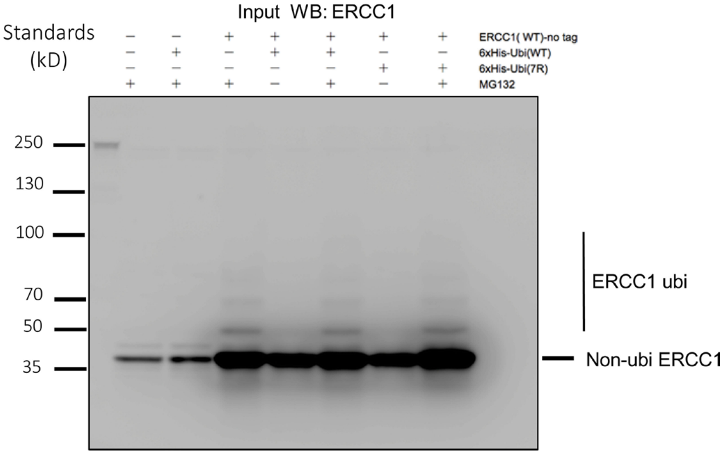

Supplementary Figure 2: Rerun combined input and immunopurified samples and whole gel images from selected parts of main Figure 1 to show the full extent of ERCC1 ubiquitination in cell lysates before and after purification and that the bottom rung of the ubiquitinated ERCC1 ladder comigrates with input ERCC1. (**A**) The MG132-exposed input and IP samples from control non-transfected A375 cells and cells co-transfected with 6xHis-tagged wild-type human ubiquitin and non-tagged wild-type human ERCC1(1-297) from main Figure 1A were run together on one gel and western blotted for ERCC1. The red arrow on the shorter exposure of the blot indicates comigration of ERCC1 from the input sample with the bottom rung of the ubiquitinated ERCC1 ladder. (**B**) Whole gel from Figure 1C Immunopurification. (**C**) Whole gel from Figure 1E Input. In both panels B and C, note the extensive ERCC1 ubiquitination ladder in MG132-exposed samples.

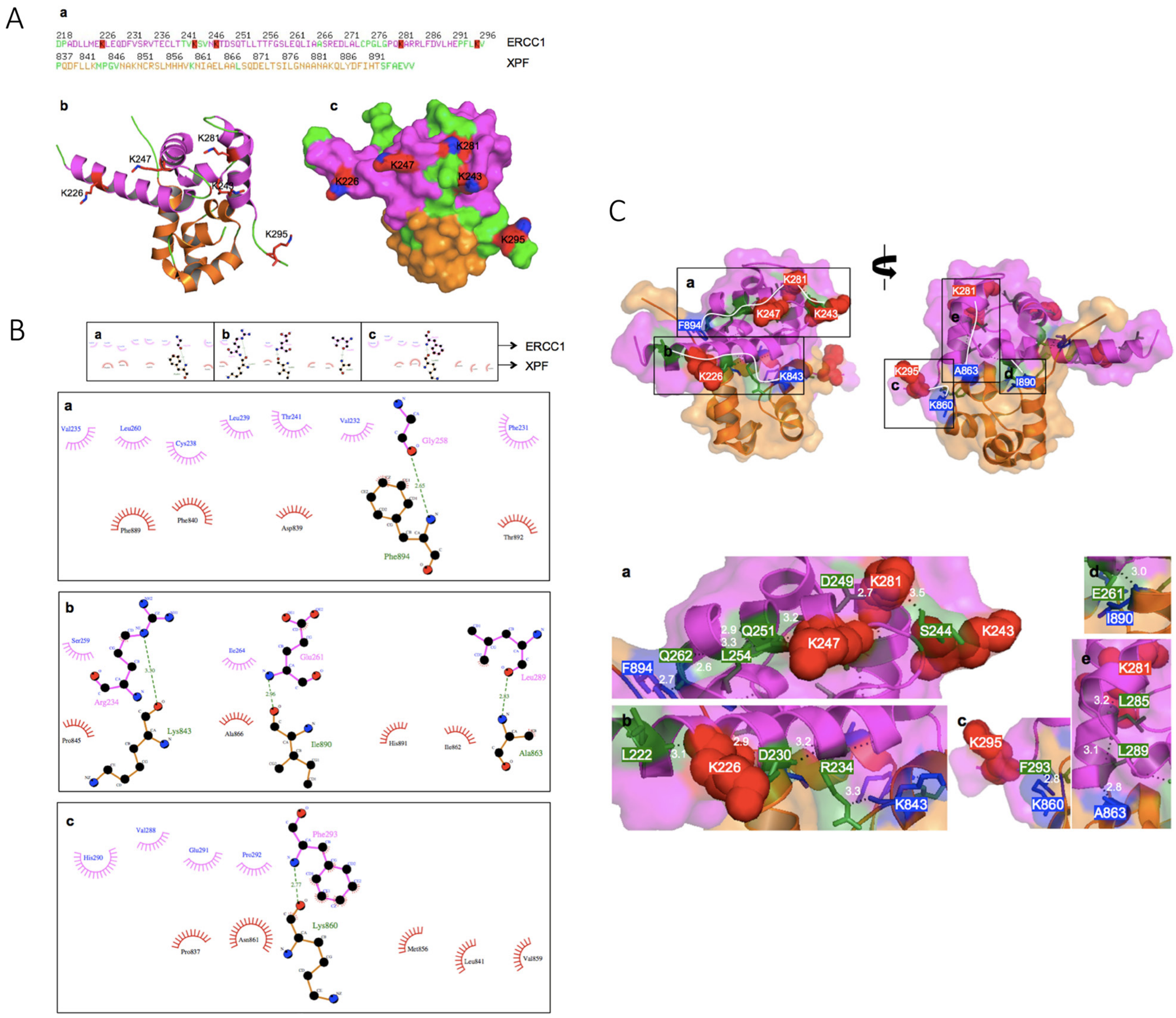

Supplementary Figure 3: Structural characteristics of lysine residues in the XPF binding domain of ERCC1. (**A**) Characteristics of the five lysines in the ERCC1 $(HhH)_2$ domain. The protein sequences of the interacting ERCC1 and XPF $(HhH)_2$ domains (a), tertiary structure cartoon (b) and surface (c) are presented. ERCC1 residues are coloured magenta while XPF are orange, with the exception of residues in loop structures (green) and ERCC1 lysines (red, with ε-amino groups marked in blue). Figure created using PyMOL v1.7.4 with the ERCC1-XPF $(HhH)_2$ domain crystal structure (PDB code 2A1J). Note: ERCC1 Lys226, Lys247 and Lys281 are in helices while Lys243 and Lys295 are in loops and ε-amino groups of all five lysines are exposed and so accessible to ubiquitination. (**B**) Interacting residues between ERCC1 and XPF $(HhH)_2$ domains. ERCC1 residues are coloured in magenta and XPF in orange. Hydrophobic interactions are indicated as brush icons and hydrogen bonds are indicated as green dashes with numbers showing their length in Å. Figure created by Ligplot+ v 1.4.5 with the ERCC1-XPF $(HhH)_2$ domain crystal structure (PDB code 2A1J). Note the five hydrogen bonds between ERCC1 and XPF residues. (**C**) Direct and indirect interactions between lysines of ERCC1 and the five residues of XPF that form hydrogen bonds with ERCC1 are shown. The basic colour of ERCC1 is magenta and of XPF is orange. The five ERCC1 lysines are shown in sphere model and red colour and the five residues of XPF are in stick model and blue colour, while the other residues of ERCC1 involved are in stick model and green colour. The white lines indicate the route of the interactions and the hydrogen bonds are indicated as dashes with numbers showing their length in Å. Figure created using PyMOL v1.7.4.

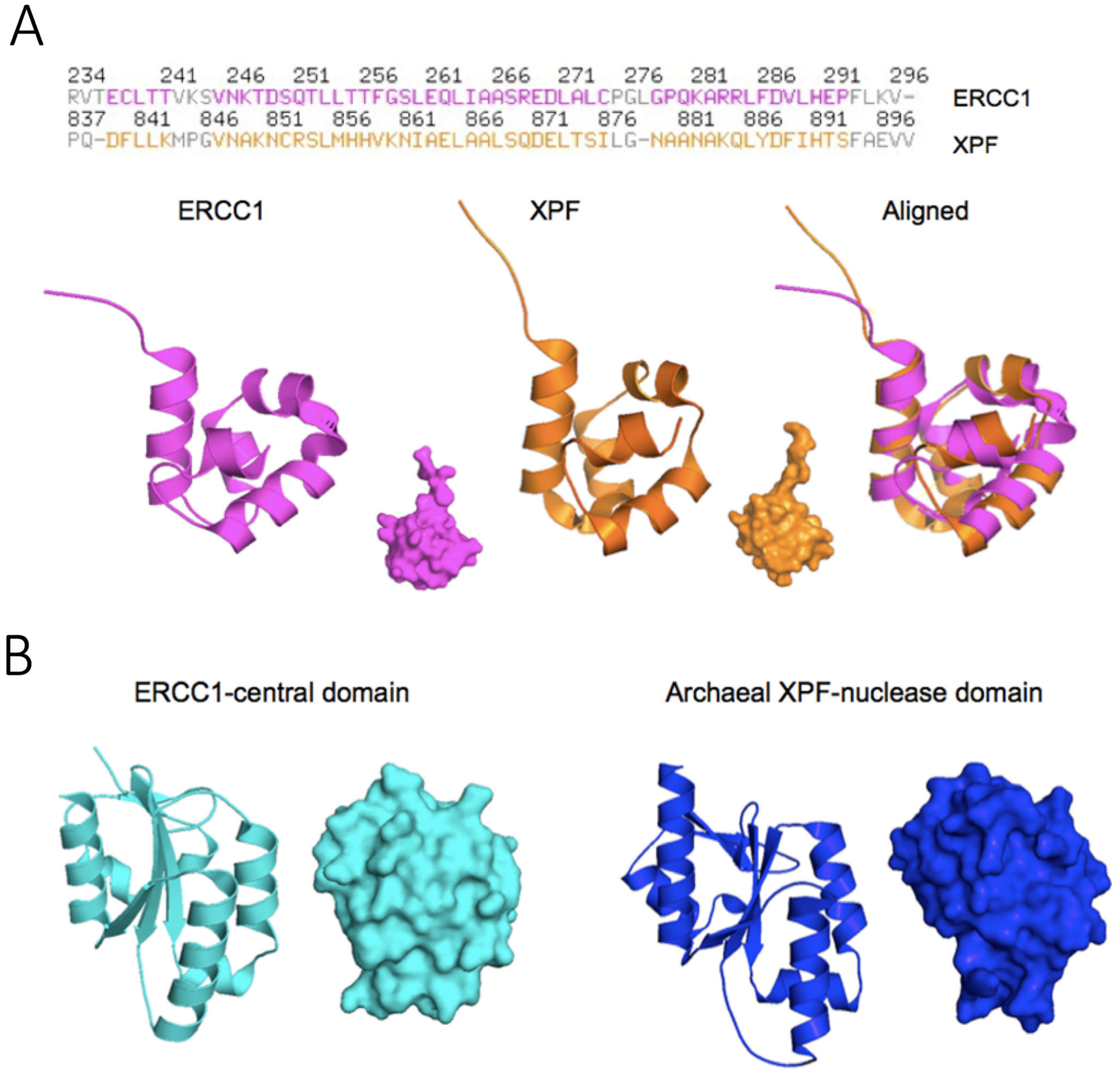

Supplementary Figure 4: Structural evidence supporting the possibility of ERCC1 homodimerization. (**A**) Similarity of the human ERCC1 and XPF $(HhH)_2$ domains. The aligned amino acid sequences are shown. Non-gray residues are in regions of high structural similarity. Secondary and tertiary structure and the overall similarity of ERCC1 and XPF are shown below. ERCC1 is coloured magenta and XPF is coloured orange. Figure created using PyMOL v1.7.4 with the crystal structure of the complex between the C-terminal domains of human ERCC1 and XPF (PDB code 2A1J). (**B**) Similarity between the central domain of human ERCC1 and the nuclease domain of archaeal XPF. Secondary and tertiary structure of the central domain of ERCC1 is coloured cyan and archaeal XPF nuclease domain is coloured blue. Figure created using PyMOL v1.7.4 with the solution structure of the ERCC1 central domain (PDB code 2JPD) and nuclease domain crystal structure of archaeal XPF/Mus81 homolog, Hef, from *Pyrococcus furiosus*, (PDB code 1J23).

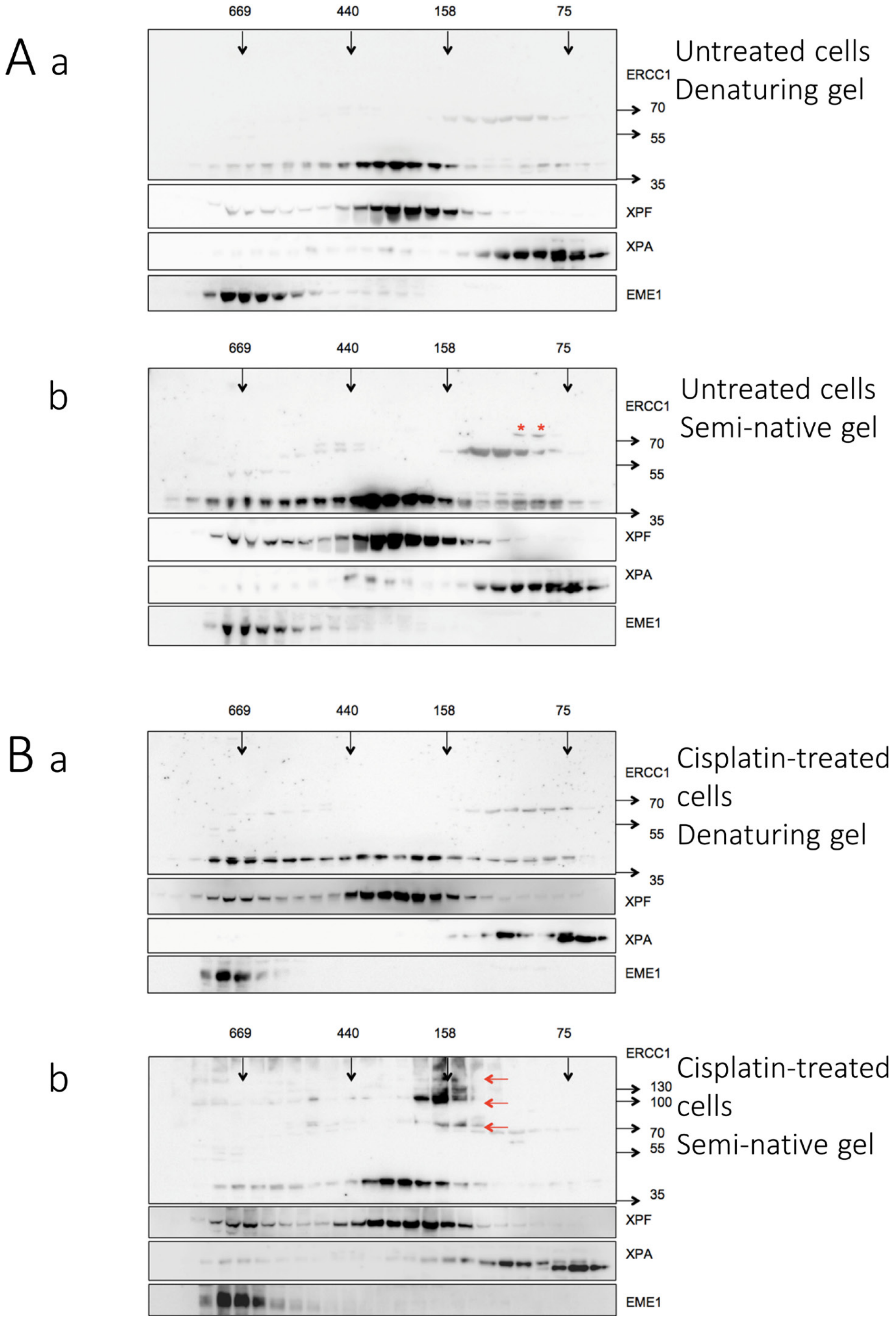

Supplementary Figure 5: Size fractionation of ERCC1-containing complexes from melanoma cells provides support for the homodimerization of endogenous ERCC1. Native lysates from A375 cells were subjected to size exclusion chromatography and size fractions were collected from the column void down to 20 kD. Each third fraction was selected for western blotting. Samples were blotted for ERCC1, XPF, XPA (NER marker protein) and EME1 (ICL repair marker protein). The positions of molecular weight markers in the size fractions are indicated across the top of the gels. (**Aa**) Normal denaturing western blot, (**Ab**) semi-native blot, both on control A375 cells. Red asterisks on the semi-native blot indicate potential ERCC1 homodimers. (**Ba**) Normal denaturing western blot, (**Bb**) semi-native blot, both on A375 cells exposed to 25 µM cisplatin for 5 h. Note on the denaturing gel the cisplatin-induced shift in the distribution of both ERCC1 and XPF away from presumed NER complexes and towards larger EMEL-containing ICL repair complexes. Note on the semi-native gel the disappearance of the potential ERCC1 homodimers in two fractions of around 80 kD seen in untreated cells and, instead, the appearance of potential ERCC1 homodimers and larger ERCC1 species in higher molecular weight fractions (indicated by the red arrows).

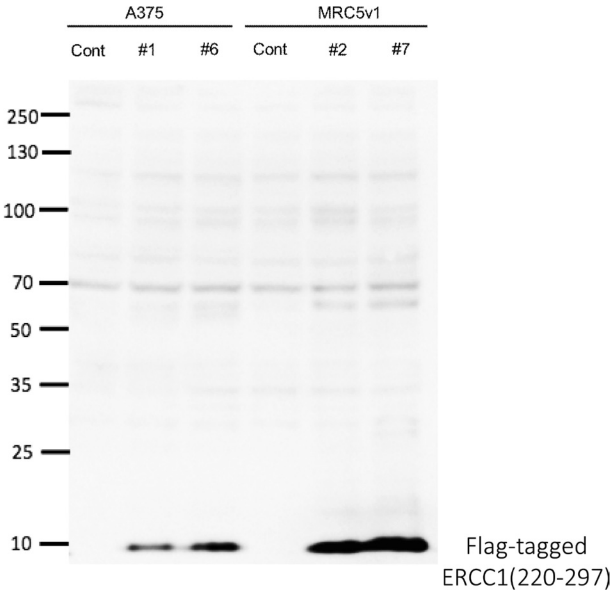

Supplementary Figure 6: Detection of Flag-tagged ERCC1 (220-297) in transfected A375 cells. Whole gel, from which the panel in main Figure 5A was taken, to show the detection of Flag-tagged ERCC1 (220-297) by the antibody to the Flag tag. Predicted size for ERCC1 (220-297) with 3xFlag tag is 11.5 kD.

SUPPLEMENTARY MATERIALS AND METHODS

ERCC1 and ubiquitin construct details

Full-length and truncated human ERCC1 coding sequences were generated by PCR using plasmid pEGFP-C1-hERCC1 as template. 5′ primers contained an EcoRI restriction site and initiator ATG, 3′ primers all contained a BamHI restriction site, some also contained a translational stop codon. Double-digested PCR fragments were cloned into the EcoRI and BamHI restriction sites of pcDNA3.1(-)/myc-His A or p3xFLAG-CMV-14. ERCC1 expressed from p3xFLAG-CMV-14 contained a C-terminal Flag tag, while ERCC1 expressed from pcDNA3.1(-)/myc-His A was non-tagged since the 3′ primer contained a translational stop codon. Primers used are shown in Supplementary Table 1.

Denaturing and semi-native gel systems

NuPAGE® LDS Sample Buffer (4×) (Life Technologies Ltd., NP0007)

NuPAGE® Sample Reducing Agent (10×) (Life Technologies Ltd., NP0004)

NuPAGE® Bis-Tris Precast Mini or Midi Gels (Life Technologies Ltd.)

NuPAGE® MOPS SDS Running Buffer (Life Technologies Ltd., NP0001)

NuPAGE® MES SDS Running Buffer (Life Technologies Ltd., NP0002)

For semi-native gels, the sample reducing agent was omitted and samples were not boiled.

In vivo ubiquitination assay details

On Day 1 A375 cells were plated in 10 cm dishes so as to be 80–90% confluent the next day. On Day 2, the required ERCC1 test plasmid was co-transfected with the 6xHis-tagged ubiquitin plasmid. On Day 3, proteasome inhibitor MG132 treatment was performed if required and then, after removing the medium and two PBS washes, cell pellets were collected by scraping. 10% of each total cell pellet was lysed with RIPA buffer in a 1.5 ml Eppendorf tube as the input sample. The remaining 90% of cells for the IP sample were lysed in the lysis buffer for the *in vivo* ubiquitin assay in a 15 ml centrifuge tube and sonicated to help break the nuclear membrane. 50 µl packed volume (100 µl supplied beads) of Ni-NTA agarose was prewashed 3 times with buffer A and incubated with each IP cell lysate on a roller mixer overnight at 4°C (or for 2-3 hr at room temperature). On Day 4, beads were spun down (2000 rpm, 3 min) and transferred to 1.5 ml screw cap Eppendorf tube. Then beads were washed sequentially with 1ml of buffers A, B, C, D and E at room temperature on a rotation table. The supernatant was checked with G250 protein dye after each spin (10 µl supernatant in 200 µl G250 in a 96-well plate), and if the dye showed no more blue colouration compared with the wash buffer alone, then washing with that particular buffer was finished (sometimes more than two washes with buffers D or E were needed). The protein pulled down by the beads was eluted with 75 µl elution buffer (30 min on rotation table at room temperature) and after centrifugation (14000 rpm, 5 min) was transferred to a new 1.5 ml Eppendorf tube for western blotting.

Stock solutions: 1M Imidazole, 0.2 M Na_2HPO_4, 0.2 M NaH_2PO_4

Buffer A (pH 8.0, 200ml): 114.6 g Guanidinium-HCl, 94.7 ml 0.2 M Na_2HPO_4, 5.3 ml 0.2 M NaH_2PO_4, 2 ml 1 M Tris-HCl (pH 8.0), 140 µl 14.3 M β-mercaptoethanol.

Lysis buffer: add 0.5 ml of 1 M imidazole per 100 ml of buffer A

Buffer B (pH 8.0, 100ml): 48 g Urea, 47.35 ml 0.2 M Na_2HPO_4, 2.65 ml 0.2 M NaH_2PO_4, 1 ml 1 M Tris-HCl (pH 8.0), 70 µl 14.3 M β-mercaptoethanol.

Buffer C (pH 6.3): 96 g Urea, 22.5 ml 0.2 M Na_2HPO_4, 77.4 ml 0.2 M NaH_2PO_4, 2 ml 1 M Tris-HCl (pH 6.3),140 µl 14.3 M β-mercaptoethanol.

Buffer D: Add 0.2 ml of 10% Triton X-100 to 10 ml of buffer C.

Buffer E: Add 0.1 ml of 10% Triton X-100 to 10 ml of buffer C.

Elution buffer (10 ml): 2 ml 1M Imidazole, 5 ml 10% SDS, 1.5 ml 1 M Tris-HCl (pH 6.7), 1 ml Glycerol, 500 µl 14.3 M β-mercaptoethanol.

Immunocytochemistry

A375 cells on coverslips were washed with PBS and fixed in 4% formaldehyde-PBS for 20 min. Cells were washed 3 times with PBS and then permeabilised with 0.5% Triton X-100 in PBS for 5–20 min before 3 further PBS washes. After being blocked in 10% donkey serum for at least 30 min, cells were incubated in primary antibody (1:200 in 10% donkey serum) for 1 h, and then incubated in secondary antibody (1:500 in 10% donkey serum) for 30 min -1 h, with 3 washes in PBS- 0.1% Tween 20 before each antibody incubation. After another 2 PBS washes, cells were stained with DAPI (1:6000 dilution of 1 mg/ml stock diluted in PBS) for 10 min. Finally, after 3 PBS washes, coverslips with cells were mounted with Vectashield (Vector Labs., H-1000) and examined using the fluorescent microscope. If a cytoplasmic marker was needed, Mitotracker staining (Life Technologies Ltd.) was used (100 nM added to culture medium for 15 min before fixation).

Immunoprecipitation

Anti-Myc Tag Magnetic Beads. A375 Cells (90%–95% confluent in a 100 mm dish) were washed three times with PBS, scraped and transferred into a 1.5 ml Eppendorf tube. Cell pellets were suspended in 1 ml cold lysis buffer (50 mM Tris-HCl pH7.5, 150 mM NaCl, 0.05% NP-40)

with Roche complete protease inhibitor cocktail and briefly sonicated (up to 10 s). After centrifugation (12000 rpm for 10 min at 4°C), supernatant was transferred to a new tube and 20 µl was taken out into another tube as the input sample. Anti-Myc-tag mAb-magnetic beads (M047-11, MBL International, Woburn, MA) were added into the remaining supernatant according to the manufacturer's instructions, well mixed and incubated on a tube rotator at 4°C overnight. Next day, beads were washed three times with wash buffer (PBS pH 7.4 with and without 0.02% Tween-20) containing protease inhibitor cocktail in a magnetic rack. The target protein bound to the beads was eluted by heating (75–95°C for 5–10 min) in 30–70 µl NuPAGE LDS Sample Buffer containing NuPAGE Sample Reducing Agent.

Protein A Dynabeads. This assay was designed for detection of linear chain polyubiquitination. 5 mM iodoacetamide was added to all the buffers just before use to inhibit deubiquitinase activity. 50 µl of Protein A Dynabeads (Life Technologies Ltd., 10001D) for each sample were washed with wash buffer and incubated in 200 µl wash buffer with 10 µl anti-linear Ubiquitin antibody at room temperature for 10–30 min, or at 4°C overnight. Other steps were performed as above.

Size exclusion chromatography

Native lysates containing 30–50 mg of total protein were made from six dishes of A375 cells (90% confluent) in a buffer (50 mM Tris-HCl pH 7.4, 0.27 M sucrose, 1% (v/v) Triton X-100, 1 mM EDTA, 0.1% (v/v) 2-mercaptoethanol) with protease inhibitor cocktail (Roche). In addition to the mild lysis buffer, sonication was used to help break the nuclear membrane since most ERCC1 is in the nucleus. Lysates were then filtered (0.22 µm) before loading onto a HiLoad 16/60 Superdex-200 (Sigma-Aldrich, 120 ml/5 ml sample containing up to 10 mg/ml protein) column in 50 mM Tris-HCl pH 7.4 buffer containing 1 mM EDTA, 0.2 M sodium chloride, and 0.1% (v/v) 2-mercaptoethanol. The column was equilibrated with XPF-ERCC1 binding buffer (50 mM sodium phosphate buffer pH 6, 500 mM NaCl and 1 mM DTT) before the protein preparation was applied. Chromatography was monitored by measuring absorbance at 280 nm and 0.5 ml fractions were collected into a DTR V3 96-well short plate from the column void down to 20 kD. Molecular weight markers (Thyroglobulin - 669 kDa, Ferritin - 440 kDa, Aldolase - 158 kDa, Conalbumin - 75 kDa, Ovalbumin - 43 kDa, Carbonic anhydrase - 29 kDa, Ribonuclease - 13.7 kDa and Aprotinin - 6.5 kDa) were used to evaluate the molecular weight of proteins present in the elution volume and molecular sizes of the protein complexes of interest were calculated from a plot of the elution volume versus the log of the molecular size.

NER assay for ERCC1 constructs in ERCC1-deficient A375 cells

To isolate an ERCC1-null A375 derivative, cells were co-transfected with ERCC1 CRISPR guide RNAs 1, 2 and 6 (GenScript, Piscataway, NJ) together with Cas9- and GFP-expressing plasmid pX458. 24 h later GFP-expressing cells were purified by FACS and plated at low density in non-selective culture medium. Surviving colonies were expanded and screened by western blotting and colonies lacking ERCC1 were identified. The clone used had no detectable ERCC1, reduced levels of XPF and NER activity < 1% of control A375 cells.

ERCC1-null A375 cells were plated in a black 96-well plate at 1.8×10^4 cells per well in 100 µl supplemented DMEM. 24 hours after plating, Lipofectamine 2000-mediated cotransfections were carried out as recommended by the supplier (Life Technologies Ltd.). 25 ng of plasmid pEGFP-C1 (Clontech Laboratories Inc., Mountain View, CA), either non-damaged or UVC-irradiated, 10 ng of pGL3 (firefly luciferase control plasmid, Promega UK Ltd., Southampton, UK), 25 ng of XPF plasmid and either 2, 0.5, or 0.125 ng of ERCC1 construct plasmid DNA with 0.75 µl Lipofectamine 2000 were used per well and added in 50 µl OptiMEM (Life Technologies Ltd.). GFP fluorescence and luminescence were measured 48 h after transfection. Fluorescence and luminescence readings from non-transfected wells were used for background correction. On each plate, 4 wells were used for each transfection and condition and the GFP/luciferase ratio was calculated for each well. The luciferase signal provides a control for transfection efficiency. The effect on NER for each of the ERCC1 constructs was determined by dividing the GFP/luciferase ratio for the damaged GFP plasmid by the same ratio for the non-damaged GFP plasmid. This value was then divided by the equivalent value for control cells where the transfections omitted an ERCC1 plasmid and NER activity was plotted as fold over this background level against the amount of each ERCC1 plasmid transfected.

Supplementary Table 1: PCR primers

Name	Sequence 5′-3′
Primers for constructing ERCC1 deletion mutants	
hERCC1-F-EcoRI	GCCCTG*GAATTC*ATGGCAAAATCCAACAGCATCAT
hERCC1-R-BamHI	GAGCTC*GGATCC*TCAGGGTACTTTC
hERCC1(297)-R- BamH1 (for p3xFlag-CMV-14)	CAGCTG*GGATCC*GGGTACTTTCAAGAAGGGCTCGT
hERCC1(97)-F-EcoRI	CTGAAA*GAATTC*ATGAAATCCAACAGCATCATTGTGA
ERCC1(220)-F-EcoRI	TATGAG*GAATTC*ATGGCGGACCTCCTGATGGAGAA
ERCC1(219)-R-BamH1	CATCAG*GGATCC*TCATGGTTTCTGCTCATAGGCCT
hERCC1(219)-R- BamH1 (for p3xFlag-CMV-14)	CAGGAG*GGATCC*TGGTTTCTGCTCATAGGCCTTGT
Primers for constructing ERCC1 single site or combination site mutants	
hERCC1(K226R)-F	GACCTCCTGATGGAGAGGCTAGAGCAGGACT
hERCC1(K226R)-R	GAAGTCCTGCTCTAGCCTCTCCATCAGGAGG
hERCC1(K243R)-F	TGTCTGACCACCGTGAGGTCAGTCAACAAAA
hERCC1(K243R)-R	CCGTTTTGTTGACTGACCTCACGGTGGTCAG
hERCC1(K247R)-F	ACTGTCCGTTCTGTTGACTGA
hERCC1(K247R)-R	ACCGTGAAGTCAGTCAACAGAACGGACAGT
hERCC1(K281R)-F	GGCCTGGGCCCTCAGAGAGCCCGGAGGCTGT
hERCC1(K281R)-R	CAAACAGCCTCCGGGCTCTCTGAGGGCCCAG
hERCC1(K295R)-R-BamH1	GATCTA*GGATCC*TCAGGGTACTCTCAAGAAG
Primers for constructing ubiquitin single site or combination site mutants	
hUbiquitin(K6R)-F	TGCAGATCTTCGTGAGGGACCCTGACTGGTAA
hUbiquitin(K6R)-R	TTACCAGTCAGGGTCCTCACGAAGATCTGCA
hUbiquitin(K11R)-F	AGACCCTGACTGGTAGGGACCATCACTCTCGA
hUbiquitin(K11R)-R	CGAGAGTGATGGTCCTACCAGTCAGGGTCT
hUbiquitin(K27R)-F	CCATTGAGAATGTCAGGGCAAAGATCCAAGA
hUbiquitin(K27R)-R	TCTTGGATCTTTGCCCTGACATTCTCAATGG
hUbiquitin(K29R)-F	AGAATGTCAAGGCAAGGATCCAAGACAAGGA
hUbiquitin(K29R)-R	TCCTTGTCTTGGATCCTTGCCTTGACATTCT
hUbiquitin(K-27,29-R)-F	ATTGAGAATGTCAGGGCAAGGATCCAAGACA
hUbiquitin(K33R)-F	CAAAGATCCAAGACAGGGAAGGCATCCCTCC
hUbiquitin(K33R)-R	GGAGGGATGCCTTCCCTGTCTTGGATCTTTG
hUbiquitin(K48R)-F	TGATCTTTGCTGGGAGACAGCTGGAAGATGG
hUbiquitin(K48R)-R	CCATCTTCCAGCTGTCTCCCAGCAAAGATCA
hUbiquitin(K63R)-F	ACTACAACATCCAGAGAGAGTCCACNCTGCA
hUbiquitin(K63R)-R	TGCAGNGTGGACTCTCTCTGGATGTTGTAGT

Note: Start and stop codons are highlighted in yellow; restriction enzyme sites are in *italics* and red; mutated bases are in red.

Supplementary Table 2: Antibodies for western blotting

Target Protein	Primary antibody	Secondary antibody
ERCC1	ERCC1 (FL297)<br>Rabbit polyclonal antibody<br>(Santa Cruz, sc-10785)	HRP-conjugated<br>Goat anti-rabbit<br>(Dako, P0217)
XPF	XPF Ab-5 (Clone 51)<br>Mouse monoclonal antibody<br>(Thermo Scientific, MS-1385)	HRP-conjugated<br>Rabbit anti-mouse<br>(Dako, P0260)
Flag tag	Monoclonal anti-Flag M2, Clone M2<br>(Sigma-Aldrich, F1804)	HRP-conjugated<br>Rabbit anti-mouse<br>(Dako, P0260)
Myc tag	Rabbit polyclonal to Myc tag<br>(Abcam, ab9106)	HRP-conjugated<br>Goat anti-rabbit<br>(Dako, P0217)
XPA	Anti-XPA (C-terminal)<br>(Sigma, X1254)	HRP-conjugated<br>Goat anti-rabbit<br>(Dako, P0217)
EME1	Anti-EME1<br>(Immuquest, IQ284)	HRP-conjugated<br>Rabbit anti-mouse<br>(Dako, P0260)
RAD51	Rad51Ab-1<br>(Thermo Scientific, MS-988-B1)	HRP-conjugated<br>Rabbit anti-mouse<br>(Dako, P0260)
β-actin	Monoclonal Anti-β-actin, Clone AC-15<br>(Sigma, A1978)	HRP-conjugated<br>Rabbit anti-mouse<br>(Dako, P0260)
linear Ubiquitin	Monoclonal anti-linear Ubiquitin<br>(Millipore, Clone 1E3, MABS199)	HRP-conjugated<br>Rabbit anti-mouse<br>(Dako, P0260)
